# Advanced Tuneable Micronanoplatforms for Sensitive and Selective Multiplexed Spectroscopic Sensing via Electro‐Hydrodynamic Surface Molecular Lithography

**DOI:** 10.1002/advs.202306068

**Published:** 2024-01-15

**Authors:** Paulo De Carvalho Gomes, Martin Hin‐Chu, Jonathan James Stanley Rickard, Pola Goldberg Oppenheimer

**Affiliations:** ^1^ School of Chemical Engineering Advanced Nanomaterials Structures and Applications Laboratories College of Engineering and, Physical Sciences University of Birmingham Edgbaston Birmingham B15 2TT UK; ^2^ Department of Physics Cavendish Laboratory University of Cambridge JJ Thomson Avenue Cambridge CB3 0HE UK; ^3^ Healthcare Technologies Institute Institute of Translational Medicine Mindelsohn Way Birmingham B15 2TH UK

**Keywords:** electro‐hydrodynamic surface molecular lithography, micronano‐substrates, surface enhanced raman scattering

## Abstract

Micro‐ and nanopatterning of materials, one of the cornerstones of emerging technologies, has transformed research capabilities in lab‐on‐a‐chip diagnostics. Herein, a micro‐ and nanolithographic method is developed, enabling structuring materials at the submicron scale, which can, in turn, accelerate the development of miniaturized platform technologies and biomedical sensors. Underpinning it is the advanced electro‐hydrodynamic surface molecular lithography, via inducing interfacial instabilities produces micro‐ and nanostructured substrates, uniquely integrated with synthetic surface recognition. This approach enables the manufacture of design patterns with *tuneable* feature sizes, which are functionalized via synthetic nanochemistry for highly sensitive, selective, rapid molecular sensing. The development of a high‐precision piezoelectric lithographic rig enables reproducible substrate fabrication with optimum signal enhancement optimized for functionalization with capture molecules on each micro‐ and nanostructured array. This facilitates spatial separation, which during the spectroscopic sensing, enables multiplexed measurement of target molecules, establishing the detection at minute concentrations. Subsequently, this nano‐plasmonic lab‐on‐a‐chip combined with the unconventional computational classification algorithm and surface enhanced Raman spectroscopy, aimed to address the challenges associated with timely point‐of‐care detection of disease‐indicative biomarkers, is utilized in validation assay for multiplex detection of traumatic brain injury indicative glycan biomarkers, demonstrating straightforward and cost‐effective micro‐ and nanoplatforms for accurate detection.

## Graphical abstract

Development of design micro‐ and nanostructured sensitive/specific, multiplexed advanced reproducible and tuneable (SMART) substrates via advanced micro‐engineering combined with molecular surface recognition. Tailor‐designed surfaces engineered via electrohydrodynamic surface molecular lithography enable the *bottom‐up* tailored micro‐ and nanofabrication with the generated platforms integrating surface enhanced Raman scattering nanoarchitectures with built‐in specific ‘nano‐roughness’.

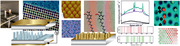

## 1 Introduction

Controllable patterning of materials via emerging lithographic techniques with precise microengineering, uniquely integrated with surface molecular recognition, can enable the translation of new knowledge into real‐world applications in the areas of nanotechnology, advanced materials and device manufacturing. These, would in turn, deliver versatile miniaturized platform technologies, underpinned by the control of the creation of functional biosensing nano‐architectures and their interactions with materials and optics to radically transform the field of point‐of‐care diagnostics. This could also advance the field of medical sensor development to a level of accuracy (≥ 90%), sensitivity (pg‐fg mL^−1^) and selectivity suitable for reliable and rapid (i.e., affordable, sensitive, specific, user‐friendly, rapid, reliable, equipment‐free, deliverable (ASSURED) criteria) identification of disease biomarkers from biofluids at minute concentrations.

The highly sensitive spectroscopic technique of surface enhanced Raman spectroscopy (SERS) has been shown to be capable of detection down to single molecule levels via enhancement of localized optical fields on metallic micro‐ and nanostructures.^[^ ^1^, ^2^, ^3^ ^]^ Thus SERS, posed as a unique optical sensing method, holds great potential for a widespread use for portable sensing and particularly for point‐of‐care diagnostics with many envisioned ramifications.^[^ ^4^, ^5^, ^6^, ^7^, ^8^, ^9^, ^10^, ^11^, ^12^, ^13^, ^14^, ^15^, ^16^ ^]^ An orchestrated advancement in micro‐ and nanofabrication of design surfaces with empirical validation would unlock its further usability as widespread analytical tool, providing the scientific community with a pathway to state‐of‐the‐art applied spectroscopic molecular systems. The instantaneous detection of target analytes at considerably lower detection limits without complex sample preparation, as well as ease of portability along with its suitability for rapid biomarkers identification, makes SERS well‐suited to address the various challenges associated with point‐of‐care diagnostics. However, whilst SERS techniques have been evolving and high‐enhancement is possible with metallic nanoparticles, for example, colloids, only a minute fraction of these, that is, “hotspot,” exhibits SERS‐activity, substantially affecting the achievable signal and sensitivity due to the large sample variability or inadequate batch‐to‐batch reproducibility.^[^ ^2^, ^17^, ^18^, ^19^, ^20^ ^]^ Furthermore, despite the remarkable progress in the last few decades where Raman instrumentation has made great strides forward, providing a route to the miniaturisation of sensing devices, nanotexturing of plasmonic hierarchical architectures via a low‐cost fabrication which simultaneously delivers reproducibility, tuneability, sensitivity and selectivity is still challenging and cumbersome. Lithographically structured micro‐ and nanoplatforms hold great promise to intrinsically overcome many of these challenges with a capability to deliver reproducible SERS measurements. Most synthetic routes to generate SERS structures to date are based on conventional patterning techniques such as photo, electron beam or focused ion beam lithographies. These are expensive, time‐consuming, bulky and often necessitate precise integration of multi‐step processes^[^ ^17^, ^18^, ^19^, ^20^, ^21^, ^22^, ^23^, ^24^, ^25^ ^]^ thus, limiting the scalability of the resulting micronano‐substrates. The limited resolution, inherent imperfections, the poor mechanical stability of the mould and pattern distortion are some of the further limiting factors of micronano lithographic and other imprinting and self‐assembly techniques. Therefore, the precise control of material features and prevention of defects, particularly in the sub‐micrometer regime, whilst producing 3D low‐cost substrates, which simultaneously fulfill the multitude criteria of high‐sensitivity, selectivity, reproducibility, tuneability and multiplicity for rapid molecular diagnostics remains a considerable challenge.

Further, a range of affinity agents including antibodies,^[^ ^26^, ^27^, ^28^, ^29^, ^30^, ^31^ ^]^ aptamers,^[^ ^32^, ^33^ ^]^ small molecules,^[^ ^34^, ^35^ ^]^ coatings,^[^ ^36^, ^37^, ^38^ ^]^ and imprinted polymer‐enabled detection of targets via gold nanoparticles on aggregation approach^[^ ^39^, ^40^ ^]^ have been explored in SERS sensing applications to enable more specific and selective detection of target analytes. Recent research in diagnostic biomarker arrays using antibodies and aptamers as biorecognition elements has reported impressive multi‐protein detection in blood serum and plasma at levels 100–1000‐fold. Many of the developed SERS substrates aimed to be readily ported for a lab‐on‐a‐chip device are based on antibodies and enzymes to specifically recognize biomarker molecules. However, often certain biomarkers do not have antibodies, and besides being expensive they and similar biological reagents, are highly susceptible to environmental conditions and can be unduly difficult to store over a long term without specialist, bulky equipment (e.g., −80 °C freezers). Furthermore, the majority of antibody‐based SERS arrays are limited to a single antibody label thus, not capable of providing the needed multiplex biomarker detection in a rapid manner. Nevertheless, early‐stage point‐of‐care diagnostics often necessitates monitoring the levels of multiple biomarkers thus, requiring a large array of antibodies in a sensor.^[^ ^41^ ^]^ Furthermore, *tuneability* to match localized surface plasmon resonance (LSPR) is not broadly available in the developed SERS systems.

Concurrently, in the past decades, surface molecular recognition methods have been evolving via modular synthetic approaches combined with molecular imprinting. These are harnessing the creation of high‐yield, complex oligosaccharide‐synthetic carbohydrate receptor assemblies and the precise production of surface‐confined templated binding sites,^[^ ^42^, ^43^ ^]^ encoding the latter with precise molecular complementarity to target oligosaccharides. By demonstrating abilities to discriminate various forms of glycoproteins and provide discriminatory information on the analytes present, these lay the platform for engineering highly selective micro‐ and nanosensor platforms. However, while the high selectivity of surface molecular recognition is well‐established^[^ ^42^, ^44^, ^45^ ^]^ for synthesizing 3D network polymers with specific binding sites for small molecules, its combination with the label‐free, multiplex and tailor‐made substrates for SERS detection has not yet been explored.

**Figure 1:**
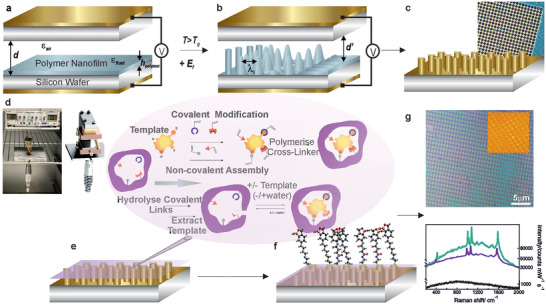
a) In the optimized micronano capacitor EHL rig, a thin polymer layer on a bottom electrode is opposed by a second electrode, creating a layered system, equivalent to a sum of two capacitors in series. b) The applied voltage gives rise to a high electric field across the dielectric material and air gap, which leads to the polarization of the dielectric polymer layer, yielding an attractive interaction between the charges at the polymer‐air interface, inducing amplified instabilities in thin nanofilm, which guide the structure formation. The dielectric discontinuity at the interface triggers the formation of displacement charges, coupling to the electric field and causing a destabilizing electrostatic pressure, which eventually overcomes the stabilizing forces of the Laplace pressure, yielding the c) energetically favorable configurations. d) The engineered EHL rig (left ‐photograph, right – schematic) incorporates actuators mounted onto a support structure and connected to the wafer stage with a built‐in vacuum chuck to hold the top electrode in‐place, suspended over the bottom electrode. This support structure is connected to a linear actuator with a travel distance of 13 mm and accuracy down to 5 nm, enabling the electrodes to be loaded and unloaded with ease and without affecting the alignment. The manufactured micro‐ and nanostructures are coated with a gold nano‐layer, generating SERS‐active substrates, which are subsequently integrated with the surface molecular recognition functionalization. e,f) The surface molecular recognition process is based on taking a cast of the target molecule on the molecularly modified EHL‐SERS surfaces to produce imprinted crosslinked thin films with synthetic receptors at nanocavities, which allow a large portion of the surface of the molecule to be recognized. In aqueous solution, these target analytes, by interacting with recognition motif monomers, for example, hydrogen bonding domains and polar residues form complexes. These are then locked down via free radical polymerization onto the gold coated EHL substrates. Target removal, subsequently, leaves synthetic receptors capable of selectively recognizing specific biomarkers of interest. The size and shape of the cavity allow the target biomolecule to occupy the cavity space, while the recognition motif orientation within the cavity binds at specific locations to the target. The interacting monomers (red, blue, and different shapes, for example, represented as triangle, circle etc.), a crosslinker and a molecule template (yellow) are mixed with the monomers assembling into a complex with the template molecule and co‐polymerize with the crosslinker, forming a highly crosslinked polymeric network around the template, which upon removal reveals complementary binding sites. This yields unique electrohydrodynamic surface molecular lithography fabricated micro‐ and nanoplatforms evident in representative optical microscopy (g, top) and AFM (g, top/inset) images of the EHSML generated SERS substrates with average SERS (not background subtracted) spectra of standard benzenethiol analyte (g, bottom) being considerably enhanced relative to the non‐patterned molecularly imprinted surfaces.

Herein, through precise control of structuring materials on a submicron scale, in an unconventional manner of inducing interfacial electrohydrodynamic instabilities integrated with synthetic nanocavities for highly sensitive plasmonics, we developed a pioneering manufacture of design micro‐ and nanostructured substrates with tuneable feature sizes. Our advanced micro‐engineering development combined with molecular surface recognition, enables *bottom‐up* tailored nanofabrication, (**Figure** **1**) with the generated platforms integrating SERS nanoarchitectures with built‐in specific “nano‐roughness.” These provide the Sensitive/specific, Multiplexed Advanced Reproducible and Tuneable (SMART) substrates. Tailor‐designed surfaces were engineered via electrohydrodynamic surface molecular lithography (EHSML), providing controllable fabrication of optimized SMART structured platforms. These act as individual detection centres for multiplexed measurements with increased sensitivity, enabling over 90% reproducibility of signal enhancement with limits of detection in the pg mL^−1^ range. Tuneability of the electrohydrodynamic lithography (EHL) nanostructures enables achieving high enhancement factors (on the order of 10^9^) by matching the excitation wavelength to local surface plasmon resonances on optically resolvable active regions. Their multiplicity and integration with surface molecular recognition enable a “lock‐and‐key” like selective SERS detection. Combining the high specificity of surface molecular functionalization, which facilitates recognition sites complementary to the shape, size and functionality of templates,^[^ ^45^ ^]^ with the high‐sensitivity offered by the EHL‐SERS, these substrates provide a powerful detection tool for biomarkers in complex matrices. SMART substrates, via reagent‐free detection of biomarkers, are subsequently validated for the detection of glycan‐biomarkers indicative of traumatic brain injury (TBI). TBI is a leading cause of morbidity and mortality worldwide with many neuro‐disabilities requiring long‐term care. Currently, there is an urgent need for new technologies to achieve timely intervention through rapid, accurate early‐stage diagnostics, when many exhibit no clinical symptoms, remaining undiagnosed until progressive phases, resulting in limited treatment options and poor prognosis.^[^ ^46^, ^47^, ^48^, ^49^, ^50^ ^]^ Molecular recognition functionalization equips the surface of the EHL SMART substrates with a stronger affinity to the biomarker (compared to other non‐target species), allowing target binding in a blood‐plasma environment. The presence of the imprinted specific binding sites introduces *built‐in* nanocavities, allowing the preferential localization of the TBI‐indicative biomarkers at the pillars, significantly enhancing the SERS signal. Thus, selectively promoting the interaction with specific biomarkers at different (color‐banded) active regions, enhances the discrimination, enabling detection down to picomolar levels for pre‐clinical diagnosis.

To enable rapid SERS data classification acquired from the optimized SMART substrates, we have further used our recently developed artificial neural network algorithm to act as a decision support tool.^[^ ^51^ ^]^ The self‐organizing Kohonen index network (SKiNET) provides a framework for multivariate analysis, which simultaneously enables dimensionality reduction, feature extraction and multiclass classification. SKiNET performs a visual separation whilst identifying the underlying chemical differences between classes, yielding simultaneously rich‐information and high‐classification specificity, even for low laser powers and short acquisition times, representative of the real‐world point‐of‐care settings. The intrinsic self‐organizing maps (SOM) of the algorithm deliver intuitive 2D clustering of the high‐dimensional spectral data according to disease state and the self‐organizing map discriminant index (SOMDI) identifies which spectral features, equivalent to the biochemical changes, are responsible for clustering. Overall, SkiNET by rapidly differentiating disease states from control groups via automated classification of the spectral data, whilst providing the assignment to a particular biomarker, lays the platform for translation of emerging point‐of‐care sensor technologies to real‐world diagnostic applications. The developed tuneable micro‐ and nanoplatforms integrate sensitive and selective multiplexed spectroscopic sensing, EHL surface molecular lithography combined with advanced spectral analysis for quantitative determination of the biomarkers. These with clinical input on the validated panels, could in the long‐term enable accurate rapid diagnostics, besides monitoring and prognostic models of neurological pathogenesis.

## 2 Results and Discussion

To fabricate the replicable and fine‐tuneable structured SERS substrates, first optimized controllable hybrid electrohydrodynamic surface molecular lithography (EHSML) was developed (Figure 1a,b), wherein uniquely, each of the individual micro‐nanostructures on the fabricated substrate yields high enhancement. This contrasts with the typical nanoparticle‐based systems for SERS, often acting as collective surfaces for signal augmentation. For patterning via the EHL, a tailor‐made high‐precision piezoelectric rig was engineered along with a dedicated top electrode to enable the optimal structural parameters for highly sensitive and consistent SERS detection of target molecules.

**Figure 2:**
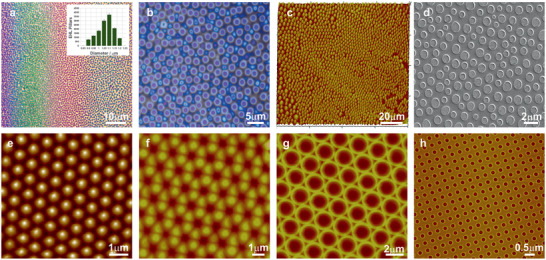
The homogeneous electric field in the EHSML capacitor with a slight wedge geometry (a) leads to a very subtle variation of 𝑑 from ≈100 nm to ≈1 µm across the sample width of 1 cm, generating bands of color corresponding to the various pillar diameters (inset) and hence, aspect ratios (Figure S2, Supporting Information). This in turn, allows not only capturing different stages of the pattern replication process (ℎ = 100 nm, V = 40 V, 𝑑 decreasing from left to right) but also, enables to optimize the tuneability establish the multiplicity of the subsequently fabricated SERS substrates. Optical microscopy (b), AFM (c), and SEM (d) images of the generated micro‐ and nanopillar structures under the homogenous Ef with a local hexagonal symmetry. Representative AFM images (e–h) of pattern formation in thin polymer films under laterally heterogeneous electric fields with respect to the parameters of the electrode, generating a range of EHSML SERS‐active high‐fidelity substrates.

EHL unconventionally induces thin nano‐film instabilities to generate a wide range of patterns on micro to nano lateral length scales.^[^ ^52^, ^53^, ^54^, ^55^, ^56^, ^57^ ^]^ The developed micro‐lithographic set‐up combines a micromanipulator with piezo actuators for the finest adjustments to enable parallel capacitor‐like patterning for consistent fabrication of sub‐micron structures (Figure 1c, inset, left). The rig comprises a copper block with a beryllium‐copper spring clamp mounted on top to hold the bottom substrate and connect it to the electrical ground. The design allows several degrees of freedom of movement and well‐aligned positioning of the top and bottom electrodes, yielding the integrity of the inter‐capacitor distance down to a nanometer scale. The top half of the rig clamps around the rectangular glass pillar using a beryllium‐copper spring. The integrated micrometre enables the micro‐adjustment of the gap between the top and bottom electrodes and the piezo actuators, the fine nano‐adjustments of the capacitor within which the polymer nano‐film is deposited. To further ensure the high precision alignment of the electrodes, an additional lithographic set‐up for dual alignment via a six‐axis hexapod actuator for the base electrode with a piezoelectric actuator mounted on a linear actuator for the top electrode was developed, yielding an alignment resolution of 5.0–30.0 ± 1.5–10.0 nm. With vacuum chucks integrated into the top and bottom alignment rig, the two electrodes could be easily mounted and brought in close proximity to each other with the actuator. These were pre‐aligned with the six‐axis positioner and finely aligned using the piezoelectric rig. The vacuum chuck ensured keeping the top and bottom electrodes in place whilst eliminating sagging under gravity at close distances. Once the top plate was brought in proximity to the bottom electrode, an initial roughing alignment down to ±200 nm was established. The top electrode was then controlled via four piezoelectric actuators in the custom‐made rig, achieving an optimal dual‐axis nano positioning down to 20.0 ± 10.0 nm. In the engineered micronano capacitor rig, the EHL process (Supporting Information S1) commenced with annealing a polymer film with initial thickness, ℎ deposited onto a bottom silicon substrate, annealed above the glass transition temperature, *T*_g_. The generated strong 𝐸_f_ at the polymer‐air interface overcomes the surface tension, amplifying film instabilities with a characteristic wavelength, 𝜆. This leads to a lateral redistribution of the film from surrounding thinning regions, further pinned to the top electrode and detached from the surrounding polymer film by draining the liquid bridge. Subsequently, resulting in the rearrangement to the energetically favorable configuration of the material, yielding the electrohydrodynamically patterned structures on the substrate. For a planar top electrode, the homogeneous electric field yields micro‐ and nanopillar formation with hexagonal symmetry (Figure 1c, inset, **Figures** 1g and **2**a–d).

The EHL fabricated platforms demonstrated consistent micronano‐structured units over the entire substrate surface areas (≈300 × 300–900 × 900 µm^2^) confirmed by the scanning electron microscopy (SEM) and the fast Fourier transform analyses (Figure S1a,b, Supporting Information). The fabricated micro‐ and nanostructured platforms were subsequently coated with a gold nanolayer (<15.0 ± 6.0> nm) with conformal and uniform Au coverage of the fabricated surfaces Figure S1e–g, Supporting Information), generating signal enhancing substrates (Figure 1c). The structures distribution at the surface, relative to the laser spot size (<diameter>: 1–2 µm), yielded consistent signal. Representative SERS spectra of a standard analyte monolayer, that is, benzenethiol, on the fabricated substrates, were collected across several areas on each substrate to demonstrate signal/substrate reproducibility. Replicability of the enhanced fingerprint signatures was established by measuring at six random locations on each substrate, fabricated via the piezoelectric EHL rig (Figure 1d,g) as well as from different substrates under identical experimental conditions. Consistent SERS signal of the analyte, retaining its stable orientation due to the optimized conformation of the underlying surfaces, exhibited a variation of <9% in relative standard deviation and <3.9% in terms of the relative peak intensities (Figure S1c,d, Supporting Information).

Subsequently, the reproducible SERS substrates were integrated with surface molecular recognition functionalization, yielding unique EHSML fabricated micro‐ and nanoplatforms for sensitive and selective biomolecular detection. This was accomplished by bio‐functionalizing the EHL‐SERS substrates with molecularly imprinted recognition sites, which are specific for a particular target molecule, delivering dedicated synthetic recognition platforms. The surface molecular recognition process (Figure 1e,f) strategy not only allows free access to binding sites for larger molecules but also offers a high specificity for such. This is crucial for improving the detection selectivity of SERS‐active substrates from complex biomatrices, particularly when aimed at detecting minute biomarker levels. In contrast to antibodies, which typically bind a single epitope, the fabricated EHSML surfaces, interacting with many physicochemical characteristics of target molecules, could offer greater discrimination between the detected biomarkers.

**Figure 3:**
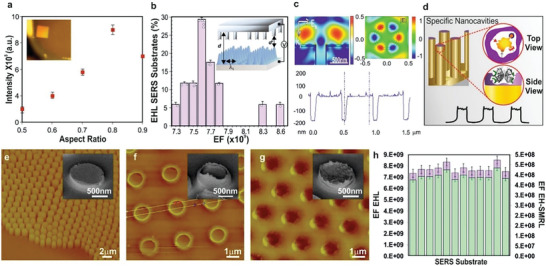
SERS intensity of benzenethiol recorded from substrates with pillars of various aspect ratios a) shows increasing aspect ratio structures result in a corresponding increase in signal up to the optimal value of 0.81 with a subsequent decrease in the obtained signal onward. (Inset) a photograph of the gold EHL‐SERS substrate (top left corner: SERS active area). b) A histogram of the electromagnetic EFs from the substrates (*n* = 17) shows a narrow distribution with a mean absolute enhancement factor on the scale of ×10.9 The EF is maximized for the red‐shifted LSPRs and dependence of the field intensity with periodic boundary conditions (c, top left) reveals increasing intensity with an increasing aspect ratio up to optimal aspect ratio at *f* = 0.81, variation mainly arising from the coupling of light into plasmon resonances which are tuned by the pillar dimensions and therefore, the relative SERS enhancement is correlated with the increased strength of plasmonic coupling into the structure. Since there is no plasmonic coupled between the adjacent pillars, a strong localization on top of each individual structure yields a high SERS enhancement. The numerically simulated optimal nanomorphology was used for fabricating the dedicated top electrode (b, inset) for EHSML with designed parameters including the inter‐electrode gap, d’, inter‐structural pitch Lm, to subsequently fabricate hierarchical micro nanostructures, under a heterogeneous electric field, with controlled periodicity rendering the adjacent pillars not plasmonically coupled to each other, generating a strong localization on top of each structure with smooth void‐like wells on top. The subsequent surface molecular recognition functionalization (d) generates the highly specific nano‐roughness, shown in the 3D cross‐sectional AFM line‐trace height profile (c, bottom), creating unique enhanced resonance in nanocavities with the whispering‐gallery plasmon modes (c, top right). Representative height and phase AFM images (e‐g) and the corresponding zoomed‐in SEM images (e–g, inset) of the e) replicable EHL fabricated SERS substrates, f) nanostructures with optimal aspect ratio and periodicity with smooth top fabricated via the dedicated top electrode and the EHSML fabricated g) molecularly imprinted micro‐nanostructured substrates with specific nano‐roughness. h) Enhancement factors from EHL‐SERS, that is, without molecular surface functionalization (green) are at least an order of magnitude higher that then EFs from the EHSML (purple) substrates (*n* = 12).

To generate specific surface confined templated nanosites a modular strategy was implemented involving four main steps: i) functionalization of a gold surface with a self‐assembled monolayer; ii) formation of a high order boronic acid‐glycan complex using compatible conditions; iii) surface‐initiated polymerization in the presence of the preformed boronic acid‐glycan complex to create specific 3D interaction sites within thin imprinted films and, iv) following formation of the well‐controlled molecular cavities, target template removal by washing with an elution buffer, owing to the reversible nature of the boronic acid/diol interactions. This approach facilitates surface binding nanosites, which are complementary to target templates in their size and shape and specific orientation. The latter recognition mode is facilitated by the initial generation of the boronic acid‐glycan complex, in which the spatial arrangement of the multiple receptors in the complex is preserved upon surface polymerization. In the EHSML, molecular surface functionalization was initially accomplished via the kinetically thermodynamic self‐assembly on top of the determined optimum film thickness (for retaining the highest enhancements) [(12–18) ± (1–3)] nm, where benzyl‐terminated self‐assembled monolayer and benzoboroxole‐terminated self‐assembled monolayer layer were formed, enabling the highest achievable SERS intensity. Two types of self‐assembled functionalizations of acrylamide‐terminated monolayer, typically used as a foundation to architect the molecularly imprinted surfaces, were used as control surfaces. First, a benzyl‐terminated self‐assembled monolayer (ST75), with a benzene ring termination, which does not interact with the target molecules (i.e., no affinity). Second, a benzoboroxole self‐assembled monolayer (ST95), which reversibly forms covalent bonds with the carbohydrate hydroxyl groups^[^ ^42^, ^58^, ^59^ ^]^ and has the same functional group as the boronic acid complex, yet without the specific conformation of the “lock‐and‐key” function (i.e., lacking the spatial specificity). Surface molecular selective recognition of glycans was subsequently established via a direct self‐assembled acrylamide terminated monolayer, a target glycan and the N’, N’‐bis‐(acryloyl cystamine)‐benzoboroxole carbohydrate complex (DSAC), which has the functional group‐specifics for the analyte of interest and anchors the whole structure to the substrate. The assembled glycan‐complex is then grafted on the self‐assembled monolayer via acrylamide co‐polymerization, affording the creation of spatially arranged sets of boronic acids on the surface that are specific to the target molecule (**Figures** 1e,f and **3**). The produced unique binding domains enable the precise covalent binding matching to the carbohydrate fragments of the target glycans. Figure 1g, top shows optical microscopy and AFM images of the EHSML generated substrates with the representative average spectroscopic SERS of standard analyte (Figure 1f, bottom), considerably enhanced relative to the non‐patterned imprinted surfaces.

The intrinsic length scales of EHL instabilities in thin fluid films under homogenous *E*_f_ are on the order of a micrometre. Imposing a *heterogeneous* electric field, smaller than the intrinsic wavelength, yields a further decrease in the length scale to sub‐100 nm feature sizes. When a laterally varying *E*_f_ is applied (via a topographically structured upper electrode) to the micro‐capacitor device, the instability is focused in the direction of the highest electrostatic pressure, which subsequently are guided toward the protruding patterns of the top electrode, forming a positive replica of the micro‐ and nanostructures (**Figure** 2).

**Figure 4:**
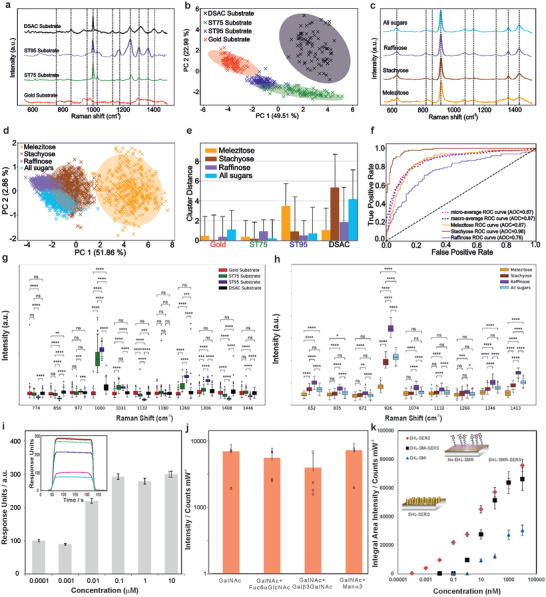
Normalized average SERS spectra of a) ST75, ST95, DSAC and gold, with the corresponding PCA analysis of the SERS spectra b) where the first two principal components of PCA show the clustering of the SERS spectra into four distinctive clusters, and of c) polysaccharides, that is, melezitose, stachyose, raffinose and their mixture with the corresponding PCA analysis d) where the loading plots of PC1 and PC2 show four clusters with a slight overlap. Euclidian distance clearly shows that DSAC has the highest cluster separation. e) Euclidian distance comparison of the PCA space between the functionalization clusters and the glycan functionalizations signify a higher specificity for ST95 and DSAC via the increased displacement distance. ROC curves representing the sensitivity versus1‐specificity f) for the surfaces molecularly functionalized EHSML substrates demonstrate high sensitivity and specificity for stachyose (AUC = 0.98, *p* < 0.0001) relative to melezitose (0.87, *p* = 0.003) and raffinose (0.76, *p* = 0.002); two‐sided *t*‐test with no multiple comparisons. Statistical analysis of the SERS spectra of the g) surface functionalization revealing the most statistically significant differences at 1000, 1031, 1260, and 1306 cm^−1^ peaks and of the h) three glycans, highlighting the statistically significant differences in SERS intensity values. From the DSAC SERS spectra for different stachyose concentrations i) the molecularly functionalized surfaces preferentially captured stachyose over the control glycans at all of the concentrations tested (SERS intensity of the assay was the strongest for range 0.1–10 µM). Representative surface plasmon resonance sensorgrams obtained for binding stachyose using it as a template on EHSML substrates with the corresponding equilibrium responses, given by the maximum response times the ratio of the concentration of injected glycan to the sum of the concentration of injected glycan and the dissociation constant, (inset) of injected stachyose. Selectivity of the EHSML generated SERS substrates for glycan detection was evaluated by comparing the results obtained from 50 µg mL^−1^ GalNAc solution with or without the coexisting components (mean values/Counts/mW) j) where the GalNAc was mixed in the presence and the absence of the coexisting glycans of Fuc6GlcNAc, Galb3GalNAc and Mana3 and subsequently subjected to SERS detection Little difference in SERS signal intensity was observed after the “interfering” components were added (two‐tail *t*‐test: ‐t Critical two‐tail < ‐t Stat < t Critical two‐tail), indicating a good selectivity for the determination of the relevant glycan markers. Error bars denote the s.t.d.e.v. k) SERS detection sensitivity of the three representative substrates of EHL‐SERS (without surfaces molecule recognition (SMR), SMR (without the EHL) and the EHSML.

The inherent EHSML versatility, enables two possible scenarios (Supporting Information S1) of homogenous *E*_f_, generating micronano‐pillars (Figure 2a–d) and heterogeneous *E*_f_, generating positive replication of imposed structures (Figure 2e–h). Building on this, we have used the former for optimizing and establishing the optimal micro‐ and nano‐structural dimension ratio of width‐to‐height for the highest SERS signal enhancement and the latter, for designing and fabricating the optimal dedicated top electrode. This not only encompasses the optimal dimensions (e.g., aspect ratio, inter‐electrode gap, inter/intra‐structural pitch) for high enhancement but also enables the creation of nanocavities, ready for the subsequent surface molecular recognition functionalization. Exploiting the micronano‐pillars with a lateral variation in aspect ratio along the wedge‐shaped cell (Figures 2a and **4**a) enables to further optimize both the tuneability as well as the multiplicity of the SERS substrates in a combinatorial manner. In the lithographic set‐up designed to allow the fabrication of SERS‐active substrates with the optimal aspect ratio, the small gap between the electrodes can be adjusted via the use of silicon oxide colloids, nanoparticles with various dimensions or lithographically structured spacers. This, in turn, tunes the achievable heights of the pillars. A broad tuneability of the structural dimensions was therefore achieved via the control of experimental parameters. The strength of the generated *E*_f_ controlled the speed, the consistency of the patterning and dictated the dominant wavelength of the instability, which in turn determined the gap between the fabricated micro‐ and nanopillars. The fraction of the film thickness to the height between the electrodes, enabled the regulation of the pillars’ diameter (Supporting Information S1).^[^ ^60^ ^]^ The planarity of the electrodes to achieve the required flatness accuracy and the alignment resolution was measured via the piezo actuators with the inbuilt strain detectors measuring the piezo displacement. This compensated for misalignment and enabled sufficient control over the flatness of the surface. Tailoring these (Figure 2a–c) allowed for the fine‐tuning of the EHSML‐SERS platforms with resulting substrates comprised of gold coated pillars with optimal aspect ratio and spacing, dictating the optical responses of the structures (Figure S2, Supporting Information). Importantly, the aspect ratio and its effect on strength of LSPR enables the tuning of SERS‐substrates for different laser excitations.

The relative SERS enhancement for the 633 nm laser excitation was found to increase strongly with the aspect ratio. Single pillars with a ratio of 0.79 yielded a nearly 30‐times enhanced signal compared to the lowest aspect ratio of 0.48. The aspect ratio continued to rise until the optimal value of 0.81 which then decreased by 12% at a ratio of 0.9 and consistently declined onward (**Figure** 3a). This was corroborated via spectrally resolved reflectance spectra across a substrate with a range of micronano‐pillars. These generated bands of color (Figure 2a) correspond to the various aspect ratios and the extinction as a function of the gap to their diameter (Figure S2a, Supporting Information). The increasing aspect ratio was accompanied by an increase in the extinction ratio, *R*/*R*_0_, arising from the enhanced coupling of light into LSPRs, tuned by the micronano‐pillar geometry thus, with that the relative SERS enhancement (Figures 1g, and 3a–c) being correlated with the increased strength of plasmonic coupling into the structure (Figures S2b and S3b, Supporting Information).

The mean absolute enhancement factor (EF) for the replicable SERS substrates with optimal dimensions (Figure 3b) was found to be 6.30 × 10^9^ at 633 nm with the confidence interval limit values, calculated according to the mean difference −1.96 × STDV (differences) and mean difference +1.96 × STDV (differences) for the SERS data (*n* = 17) was found to be (4.20 × 10,^9^ 8.40 × 10^9^). Numerical COMSOL simulations (Figure S3a, Supporting Information) revealed an enhanced electromagnetic field primarily localized at the apex of the micro‐nano gold‐pillars (Figure 3c, top left), which subsequently directed the fabrication of the optimal EHSML SERS substrates.^[^ ^54^ ^]^ While the fabricated structures couple light into plasmon‐polariton and propagate LSPRs by diffraction, the controlled periodicity renders the adjacent pillars not plasmonically coupled. This generates a strong localization at the apex, and therefore high electromagnetic field and signal enhancements resulting in red‐shifted apex plasmons. Uniquely, these arise solely from a single pillar, each of which can act as an individual detection centre in the miniaturized sensing platforms.

Establishing the highest LSPR intensity due to plasmon coupling, at the aspect ratio of 0.81, which could be tuned by variation of the periodic topologies and spacing and for which SERS signals can be achieved from concentrations as low as ppb, led to the subsequent design of a dedicated top electrode. This comprised the optimal dimensions for high EF and for the creation of specific surface molecularly imprinted nanocavities (Figure 3b, inset and Figure 3d). Exploiting the dedicated structured top electrode within the micro‐rig yields hierarchical SMART structures, with optimal EF, whilst enabling a platform for the inherent highly specific “nanoroughness” via the subsequent surface molecular recognition (Figure 3c,d). This establishes a continuous straightforward EHSML process, starting with the generation of replicable EHL‐SERS substrates (Figure 3e), through the optimal aspect ratio for highest EFs via a dedicated engineered electrode (Figure 3f), comparable to the electrofluidynamically patterned structures,^[^ ^57^ ^]^ and completed with the fabricated micro‐nanostructured molecularly imprinted substrates (Figure 3g). The latter induces a resonating spectrum, where a coupling effect is achieved via the combination of plasmon and whispering‐gallery resonances trapping and enhancing the signal. This aligns with the sphere segment void structures,^[^ ^61^ ^]^ for which it was shown that plasmons can be locally trapped to produce significantly enhanced optical fields at precise locations, where charge distributions on the walls of the cavity provided the electric field distributions constituting the plasmon modes. The fields were found to be concentrated inside the dielectric cavity and the field enhancement depending on the contribution of both reradiation and absorption loss, yielding longer plasmon confinement times. Therefore, stronger SERS excitation of molecules with larger, micron‐diameter, substrates supports even higher‐order plasmon modes.^[^ ^61^ ^]^ The fabricated unique optical nanocavities confine light to small volumes by resonant photon circulation yielding sensitive, selective and coherent SERS detection (Figure 3c, right and Figure 3h).

**Figure 5:**
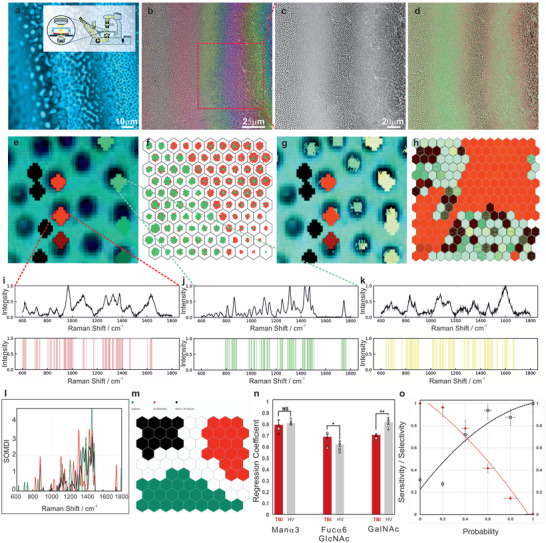
a) In situ monitoring of the EHSML patterning in the inverted optical microscope with a transparent top electrode allows observing the instability sweeping across the sample, revealing different pattern evolution stages, from early undulations with a characteristic wavelength (right) to pillars spanning the micronano‐capacitor gap. b) Representative optical microscopy and c) zoomed‐in images of EHSML fabricated tuneable substrates with a slight variation in structures’ height between the adjacent areas coupled with surface molecular functionalization of two target molecules of d) GalNAc and Fuc6GlcNAc on the different color‐banded region on the micro‐nanopillars with the corresponding duplex detection of e) GalNAc (red) and Fuc6GlcNAc (green) SERS map, overlaid over the corresponding optical image, reconstructed using representative peaks of the two components attached to adjacent active regions. h) SERS map reconstructed using the sum intensities of the distinct representative peaks of GalNAc (red), Fuc6GlcNAc (green) and stachyose (yellow) with no signal observed from in‐between the structures (black) multiplex detection. The corresponding SOM spatial clustering of SERS spectra (*n* = 300) of f) GalNAc and Fuc6GlcNAc and h) GalNAc, Fuc6GlcNAc and stachyose, where the SERS signal comes only from the EHSML micronano‐structures, and no SERS signal is observed from in‐between the pillars. Characteristic barcodes derived from the corresponding average fingerprint spectra of i) GalNAc, j) Fuc6GlcNAc, and k) Mana3 with l) SOMDI extracted features from SOM m), highlighting the most influential Raman peaks for each glycan biomarker with specific bands highlighted at 832, 967, 1085, 1272, 1325, 1380, 1431, 1467, and 1635 cm^−1^. n) A statistically significant difference in the calculated regression coefficients is found in the contribution of GalNAc (p** < 0.01) and Fuc6GlcNAc (p* < 0.1) in differentiating between the traumatic brain injury (TBI) and healthy volunteer spiked samples. Two‐sided Student's *t*‐test, *n* = 33 samples, error bars indicate the standard error. o) At a probability cut‐off point of 0.42 both the sensitivity and selectivity for this combination of markers is high with a discrimination power of 84%.

Interestingly, whilst the synergy between whispering‐gallery‐mode and surface plasmons,^[^ ^62^ ^]^ which can be tuned by altering the diameter or height of the SMART micro‐ and nanoarchitectures and the excitation laser wavelengths, yields an additional SERS enhancement in the EHL substrates, the subsequent surface molecular imprinting results in a reduction of the achievable EFs by an order of magnitude, albeit with an increased selectivity (**Figure** 4g–i). For the EHL‐SERS substrates, the calculated absolute EFs, normalized by the spot size (i.e., the illuminated area) and measured at nine random areas across each substrate, lie between 4.20 × 10^9^ and 8.40 × 10^9^ and for the EHSML substrates, typical values of EFs are on average 3.9 × 10^8^ The lower sensitivity for the EHSML surfaces is due to the fact that SERS is a distance dependent effect, known to decay away from the surface.^[^ ^63^ ^]^ Detection at 5 nm and beyond has been demonstrated at 40% of the maximum signal, and stronger SERS has been observed for >10 nm, as shown empirically by Kumar et al.^[^ ^63^ ^]^ Given that an average thickness layer for molecular recognition of 12 nm was used in our protocol, the successful detection at 75% of the maximum SERS signal combined with the nearly fivefold increase in selectivity (Figure 4) (where the presence of the imprinted specific binding sites allow the preferential binding of the target molecule), further enhanced via the inherent tuneability and multiplicity, (**Figure** **5**) renders the EHSML fabricated substrates highly sensitive and specific for rapid detection of analytes of interest at *minuscule* concentrations. Additionally, a low amount of non‐specific binding from other, not too dissimilar molecules, does not pose an issue with the EHSML since the fabricated substrates exhibit a higher selectivity for the target biomolecules.

Initially, the selectivity of the EHSML generated DSAC scaffold in comparison to the benzoboroxole‐terminated and the benzyl‐terminated self‐assembled monolayers and the planar gold substrates was evaluated. Representative SERS fingerprints for ST75, ST95 and DSAC, the molecular surface functionalization, (Figure 4a) reveal characteristic peaks of 1000 cm^−1^, associated with ν(C−C) aromatic ring vibrations for all three functionalizations, the 1031 and 1260 cm^−1^, attributed to ring deformation and CH_2_ wagging mode, respectively for ST75 and ST95 and the 1306 cm^−1^, assigned to the CH_2_ deformation vibration modes νCH_2_, for the DSAC only.^[^ ^64^ ^]^ The functionalized EHSML SERS substrates were subsequently used to establish the specific fingerprint spectra and quantitatively determine carbohydrate affinity using PCA analysis of the different surfaces before and after immersion in the stachyose solution. It is evident that the overall PCA cluster separation, that is, the Euclidean distance between mean cluster positions, changes more significantly for the DSAC functionalization with a more dispersed cluster relative to the ST75 and ST95 (Figure 4b). This is due to the surface molecular imprinting accomplished via this step, which creates a specific recognition site for the target molecule, that is, stachyose. Subsequently, the specificity of ST75, ST95, and DSAC for detecting stachyose relative to the two structurally related glycans, that is, raffinose and melezitose as well as a mixture of all three sugars, was evaluated.

The average spectra of each carbohydrate and its mixture are shown in Figure 4c. The representative spectra are dominated by several bonds. These are attributed to the C─O, C─C─O, C─C─C bending vibrations (652 cm^−1^), C─H deformation vibration, C─O─H bending vibrations (835, 872, and 926 cm^−1^), C─H and C─O─H bending vibrations (1074 and 1268 cm^−1^), C─O and C─O─C stretching (1132cm^−1^), CH‐ and OH‐ wobble vibrations (1346 and 1413cm^−1^).^[^ ^65^, ^66^, ^67^, ^68^ ^]^ The score plot of the PCA of an unsupervised clustering tendency reveals well separated clusters for stachyose, raffinose, melezitose with an overlapping cluster for the glycan mixture of the three sugars, based on the first two PCs (Figure 4d), where the Euclidean distance between the initial cluster and the cluster position following the immersion in the glycan solution indicates presence or absence of a particular sugar on the EHSML substrate. PCA of ST95, which is selective toward glycans, shows a mildly increased affinity for melezitose (3.9‐times) and stachyose (1.8). The Euclidian distance between the clusters clearly shows that DSAC, specific to stachyose, has the highest cluster separation with an increased (5.7‐fold) cluster separation to stachyose (Figure 4e). We further used receiver operating characteristic (ROC) curves, plotting the true‐positive rate against the false‐negative rate, to assess the SERS profiling data and analyze the ability of DSAC to differentiate stachyose from other sugars, that is, melezitose and raffinose, by calculating the area under the curve (AUC) for each glycan (Figure 4f). From the ROC curves, we determined the intrinsic discrimination accuracy for raffinose (AUC = 0.76), melezitose (AUC = 0.87) which did not reach a similar level of statistical significance to stachyose for which a statistically significant classification was found (AUC = 0.98). The ROC analysis shows the high sensitivity and specificity of the DSAC for stachyose relative to the two glycans, melezitose and raffinose. The PCA‐QDA classification yielded a sensitivity of 99.0%, a specificity of 98.0% and an overall accuracy of nearly 99.0% for detecting stachyose. The above results demonstrate that the specific surface molecular functionalizations, for example, the benzoboroxole in ST95 and the molecular imprinting in the DSAC, enable selective detection and discrimination of glycans, with the latter being highly specific for stachyose.

From the spectroscopic profiling and the corresponding PCA analyses, the most dominant spectral changes, determined to be statistically significant for discriminating the different functionalizations, arise from bands at 856 cm^−1^ (p^***^ < 0.0001 for ST75, ST95 and 0.001 < p^*^ < 0.01 for DSAC), 1000 cm^−1^ (p^**^ < 0.0001 for ST75, ST95, and DSAC), 1031 cm^−1^ (p^***^ < 0.0001 for ST75 and ST95), 1260 cm^−1^ (p^***^ < 0.0001 for ST75 and ST95) and 1306 cm^−1^ (p^***^ < 0.0001 for ST95), establishing these as barcode fingerprints (Figure 4g). Despite the noticeable differences in the average spectra of the molecularly functionalized surfaces, as indicated by the peaks marked by dashed lines (Figure 4a) at 774, 854, 972, 1132, 1180, 1364, 1408, and 1446 cm^−1^, these bands exhibited a lower statistical significance relative to the other peaks identified from the spectral data. DSAC spectra are found to exhibit a relatively high variance relative to gold control substrate and the ST75 and ST95 functionalizations, due to the more complex molecular surface nature of the imprinted complex. Interestingly, in this case, most of the representative peaks have no statistical significance (*p* > 0.05) to discriminate between the DSAC and gold, with the only identified peaks for the differentiation of DSAC from ST95, ST75 and gold found to be at 856 and 1000 cm^−1^.

Box and whisker plots reveal statistically significant differentiation of the three sugars identified from the majority of the spectral peaks, arising from the SERS intensity differences, which was further confirmed via the normalized intensity plots (Figure S4, Supporting Information). Whilst the peaks at 652and 1346 cm^−1^ show no statistical significance in discriminating between stachyose and a mixture of the three glycans with a variance of significance levels for peaks at 835, 872, 1074, 1132, and 1268 cm^−1^, the most intense bands at 926 and 1413 cm^−1^, were found to be statistically significant (p^***^ <0.0001). This is in correspondence with the PCA results (Figure 4d), where the clusters are primarily separated via the intensity variation. Notably, the intensity of the identified peaks is constantly shifted in a similar manner, with *I*_(melezitose)_ < *I*_(stachyose)_ ≈ *I*_(mixture of sugars)_ < *I*_(raffinose)_ for the various peaks (Figure 4h), which is also reflected in the distribution of clusters in the PCA space, strongly indicating the PC1's primarily cluster separation being based on the spectral intensity differences. This demonstrates the feasibility of our approach, where given the characteristic feature of a glycan, that is, multiple hydroxyl groups or BAs anchored at appropriate positions in the recognition cavities interact synergistically with a target molecule, promoting improved affinity and selectivity.

Surface plasmon resonance spectroscopy analysis revealed a nearly ninefold increase in the relative binding affinity for the target analyte with the stachyose‐binding nanosites (maximum binding capacity of 0.27 ± 0.03 ng mm^−2^). Only negligible or no binding affinity for the non‐target oligosaccharides (Figure 4i) due to the higher affinity from a more specific selection was demonstrated via the synthetic recognition system. Similarly, by creating raffinose or melezitose binding scaffolds, the selectivity is reversed and instead, the recognition sites only bind the chosen target saccharides.

Subsequently, the fabricated SMART EHSML platforms were assessed to detect TBI‐indicative glycan biomarkers, that is, GalNAc, Fuc6GlcNAc, Gal*β*3GalNAc and Man*α*3, first individually and then from a mixture. The chosen glycans have been recently measured from plasma and saliva samples with significant concentration changes post‐injury, which may relate to their excretion into the circulation following increased metabolism from damaged neurons,^[^ ^59^ ^]^ as indicators of destruction of nerve tissue, signposting these as biomarkers for detecting early TBI onset. Following the molecular peak signatures corresponding to specific structural and composition information, blood plasma samples of healthy patients were spiked with the TBI‐indicative glycans (Figure S5, Supporting Information) and analyzed with and without the spiked biomarker to test the recovery. Recovery observed for the spike was nearly identical, within the experimental error, to the recovery obtained for the analyte prepared in standard diluents, validating the sample matrices for the detection assay.

To evaluate the selectivity of the EHSML generated glycan‐binding of GalNAc, (Figure 4j) the scaffolds were mixed with (and without) the coexisting glycans of Fuc6GlcNAc, Gal*β*3GalNAc and Man*α*3 and subsequently subjected to SERS detection (*n* = 9). The selectivity of the substrates was evaluated by comparing the results obtained from 50 pg mL^−1^ GalNAc solution with or without the coexisting components, yielding mean values of 12 879.4 counts mW^−1^ for GalNAc only, 9293.5 counts mW^−1^ for GalNAc+Fuc6GlcNAc (p^*^ < 0.003), 8184.1 counts mW^−1^ for GalNAc+Galβ3GalNAc (*p* < 0.1) and 8453.5 counts mW^−1^ for GalNAc+Manα3 (*p* ^**^ < 0.009). Little difference in Raman signal intensity of the SERS assay was observed after the “interfering” glycans were added (the data was tested by two‐tail *t*‐test: *‐t* Critical two‐tail < *‐t* Stat < *t* Critical two‐tail), indicating good selectivity for the determination of the relevant target glycan biomarker. These results indicate that an increased selectivity in the optimized electrodynamically fabricated molecularly imprinted nanocavities enables to distinguish the difference between target and nontarget glycans, establishing their suitability for selective and sensitive biomarker recognition detection.

A comparison of three representative substrates of EHL‐SERS (without SMR), SMR (without the EHL) and the EHSML (Figure 4k) reveals the highest achievable signal from EHL‐SERS substrates, capable of analyte detection down to sub pM levels. The EHSML substrates, with an increased selectivity, exhibit a reduction in the signal enhancement by an order of magnitude (Figure 3h), yielding target analyte down to nm concentrations. Interestingly, the only molecularly imprinted substrates, with no EHL micro‐nanostructures, also exhibit a moderate signal enhancement capable of detection down to µm levels. This can be due to the fact that these substrates have a higher selectivity for the targeted biomolecules, thus attracting and concentrating them at the sensing surface, which with the underlying thin gold nano‐layer, yields the increased SERS signal.

The overarching goal of many miniaturized detection technologies under development, particularly for point‐of‐care sensitive detection, is to identify early‐stage disease via sensing of low concentration biomarkers within complex biofluid matrices. However, these often contain numerous other compounds with the overall detected signal being a superposition of the multiple target analytes. EHSML enables further addressing of this via the fabrication of SERS substrates with lateral distribution of structures’ aspect ratios. These, generating bands of color, easily observable under optical magnifying lenses (Figure 5a and Movie S1, Supporting Information), enable the inherent tuneability of the extended LSPR and the substrates for multiplex detection of target molecules. Each individual band in the micro‐ and nanostructured array can be specifically functionalized for different target molecules. Given that the measured vibrational SERS signals are spatially separated and do not overlap, enables simultaneous multiplexing of several analytes. For the detection of multiple analytes, we have fabricated tuneable arrays (with an average width of each 27 ± 3.3 µm) with a slight variation in structures’ height between the adjacent areas with pre‐determined (via the EHL physical principles) dimensions predominantly of 0.9–1.2 µm diameter each and a 1.9–3.0 µm pitch between the individual pillars (Figure 5b,c). A thin gold layer was deposited onto the micronano pillar‐based structures, which were subsequently coupled with surface molecular functionalization of different target molecules on each color‐banded region on the micro‐nanopillars (Figure 5d). Initially, for proof‐of‐concept duplex detection, two target molecules of GalNAc and Fuc6GlcNAc were immobilized in each given spatial region, which could be identified given their position on the substrate in the color band. These, bound at their corresponding spatial positions allowed the selection of the target biomarkers from the background. GalNAc and Fuc6GlcNAc were clearly detected using SERS mapping due to their different spectral signatures (Figure 5d–g). The weaker signal from Fuc6GlcNAc relative to the GalNAc could be attributed to their SERS cross sections, that is, the “chemical effect.” Representative SERS maps, overlaid over the corresponding optical images, reconstructed using the sum intensities of the distinct representative peaks of the two components show duplex detection with distinct peaks at 967, 1085, 1325, 1380, and 1635cm^−1^ of GalNAc (red) compared with and Fuc6GlcNAc (green), which has prominent peaks at 1467 cm^−1^ (*δ*(CH_2_), C‐H and N‐H wagging on the NAc group), 1431 cm^−1^ (C‐H wagging mode), 1376 cm^−1^ (*ω*(CH_2_)), 1346 and 1272 cm^−1^ (*τ*(CH_2_) Glc) associated with CH_2_ and COH deformations. For the fingerprint region, peaks at 1138, 1058 cm^−1^ are *ν*(CO)*endo* and *δ*(COH), respectively and at 832 cm^−1^ attributed to *τ*(CH_2_) as well as the ring modes with further fingerprint peaks at 1310, 863 and 808 cm^−1^ from the OH on the Glc and C‐H deformation, C‐H stretch on the NAc group and the CH and OH modes on the Glc, respectively.^[^ ^65^, ^66^, ^67^, ^68^ ^]^ The detected SERS signal arises solely from the EHSML pillars with no signal observed from in‐between the structures (black) (Figure 5e,g). Subsequent multiplex detection of GalNAc (red), Fuc6GlcNAc (green) and stachyose (yellow) was accomplished in a similar manner (Figure 5g). This demonstrates an overall approach of acquiring the SERS‐fingerprint of a set of substances and comparing it with the fingerprint vibrational spectrum of an unknown mixture, which enables to elucidate the composition of the sample and resolve the interference of various solutes in biofluids (Figure 5h).

To evaluate the discrimination between the biomarkers within the spiked blood plasma samples of healthy patients with the TBI‐indicative glycans, standard SERS spectra of the respective pure GalNAc, Fuc6GlcNAc and Manα3 (Figure S5, Supporting Information) and further glycans (Figures S5 and S6, Supporting Information) were acquired, generating specific fingerprints for each. These were barcoded via the representative SERS peaks (Figure 5i,j and Figure S5, Supporting Information) of the highest detected intensity and spectral differences, subsequently used to identify the target analytes, comparing between the TBI and control samples (Figure 5n). Further to the average SERS spectra measured using the 633 nm excitation laser spanning the fingerprint regions where distinct bands at 863, 1232, 1325, 1380, 1431, and 1467 cm^−1^ were dominant from SOMDI analysis (Figure 5l), subtle changes in spectral features were identified via SKiNET with a significant change in the ratio of peaks at 1635/1310 (O‐H stretch in GalNAc/OH on the Glc and C‐H deformation in Fuc6GlcNAc), the ratio of 1270 and 1105 cm^−1^ (O‐H bonds twisting in Man*α*3/ring breathing modes in Fuc6GlcNAc) and the 1456 cm^−1^ in GalNAc (C‐H modes on the acetyl group)^[^ ^65^, ^66^, ^67^, ^68^ ^]^ identified with SOM distinguishing between GalNAc, Fuc6GlcNAc and Manα3 (Figure 5m) with the overall SOMDI extracted underpinning spectral features from SERS spectra (Figure 5l) being responsible for the clustering observed in SOM (Figure 5m). This establishes the multiplex barcoding of a panel of biomarkers from a complex biological matrix based on their distinct SERS signatures combined with the computational SkiNET algorithm for rapid classification, via the key‐features from the spectral analysis visually represented in the coloured Raman maps, providing a selective and sensitive method for detection of TBI biomarkers.

The regression coefficients for the GalNAc, Fuc6GlcNAc and Manα3 (Figure 5n) provide further insight into the separation of detected classes, with a larger coefficient indicating a greater contribution to the spectra. A statistically significant difference is found, via one‐way ANOVA analysis, in the contribution from GalNAc, which is linked to the dominant bands at 1380, 1325, 1080 and 967 cm^−1^ associated with the CH deformation on the Acetyl group, the C‐H mode on the Gal's CH_2_OH group, the C‐H deformation on the ring and the CH modes on the Gal, respectively (Figure 5l,m) in TBI versus controls. Further peaks were identified as strong SOMDI weights as derived from the analysis provided by SkiNET (Figure 5l,m). A statistically significant change is also observed for Fuc6GlcNAc (*p* ^*^< 0.1). However, there is no statistically significant difference for Manα3, where small coefficients were fitted for TBI and controls.

It has been reported by Mondello et al. that the changes seen in serum glycans can be partially attributed to the biosynthetic and metabolic crisis after TBI, where in response to acute brain injury, multiple mechanisms consequence in mitochondrial dysfunction and increased oxidative stress with a shift from aerobic to anaerobic metabolism in neurons.^[^ ^69^ ^]^ GalNAc, playing a key‐role in cell metabolism and signalling, has been further shown by the same researchers to compromise the blood brain barrier, triggering the metabolic disruption and the biochemical cascade following TBI cell damage, resulting in its release. Mondello et al. have also recently identified a link between the observed alterations in the glycosylation biosynthetic pathways and the impact on the white matter damage and repair, and thereby clinical outcome, by assessing serum levels of 94 N‐glycans post TBI.^[^ ^69^ ^]^ An accumulation of ganglioside in the region of injury has also been demonstrated. It was further suggested that the serum glycan patterns may reflect distinct pathobiological pathways linked to different types of brain damage, that is, diffuse axonal injury versus those with mass lesions. This indicates that N‐glycan branching regulates oligo‐dendrogenesis, promotes myelination and myelin repair as well as employs pleiotropic effects in the brain with alterations shown to affect neuroinflammatory responses, neuronal excitability and promote neurodegeneration^[^ ^70^, ^71^, ^72^ ^]^ and low serum levels of GlcNAc are associated with demyelination and axon damage.^[^ ^73^, ^74^ ^]^

Monitoring the characteristic peaks identified for each glycan biomarker via the progressive sample dilutions reveals a linear relationship between SERS intensity and biomarker concentration (1 pm to 1000 nm). The goodness‐of‐fit for the linear regression model of 0.909, 0.891, and 0.853 for GalNAc, Fuc6GlcNAc and Manα3, respectively (Figure S7, Supporting Information and Figure 5o) enabled the determination of limit of detection at a predetermined laser‐power. The C‐H mode on the Gal CH_2_OH group and the corresponding deformation on the Acetyl group of peaks at 1325 and 1380 cm^−1^, the C‐H and N‐H wagging vibration mode and the C‐H stretch on the NAc group at 1431/1467 and 863 cm^−1^, the O‐H and C‐H on *mannopyranosyl* side and the O‐H and C‐H bonds vibration perpendicular to *z*‐axis at 1232 and 1464 cm^−1[^ ^65^, ^66^, ^67^, ^68^ ^]^ for Manα3 were selected as sharp peaks for GalNAc, Fuc6GlcNAc and Manα3, respectively. For deriving the limit of detection (LoD), it was calculated as three times the ratio between the standard deviation of the lowest measured concentration and the gradient of the calibration curve and the limit of quantification, expressed as 10 times the same ratio as the LoD. The determined LoD was 0.507 pg mL^−1^ (2.27 nm) and LoQ = 1526.5 pg mL^−1^ for GalNAc, 113.49 ng mL^−1^ and LoQ = 343.47 ng mL^−1^ (198.8 nm) for Fuc6GlcNAc. The calculated values without the molecular functionalization on the EHL‐SERS substrates with the lowest detectable concentration were 0.12 and 3.6 nm for GalNAc and Fuc6GlcNAc, respectively. Therefore, whilst the selectivity for molecularly functionalized surfaces was improved ninefold compared to non‐imprinted EHL‐SERS substrates, the LoD for the EHSML was on average 33 times lower than for the EHL‐SERS substrates. From assessing the EHSML substrates variation in measured SERS signal for each unit change in concentration with the ability to discriminate between the target analyte and other constituents in the sample, the overall sensitivity and selectivity are found to be inversely proportional (Figure 5o) with the optimum cut‐off of 0.42. This can be used as an initial value for the development of exploratory pre‐clinical studies ensuring the ROC curves estimation of the primary outcome with sufficient accuracy to determine the clinical cut‐off values, (guided by the threshold which maximizes the value of Youden's J statistics) which can subsequently, be used to discriminate between traumatic brain injury and healthy controls.

The developed SMART substrates were further verified within the home‐built miniaturized Raman setup, providing distinct advantages in delivering an affordable, portable and non‐invasive neurotrauma‐indicative sensing device. The miniaturized Raman system for multiplexed analyses of biomarkers comprises a spectral detection assay with a disposable integrated optofluidic SERS unit, a portable Raman system for PoC sample analyses, consisting of a laser, lenses, gold and diachronic mirrors with an echelle spectrometer (Figure S8, Supporting Information)

Finally, further to the production of 3D low‐cost substrates, which simultaneously fulfill a multitude of criteria of high sensitivity and selectivity, reproducibility, tuneability and multiplexicity, the EHSML poses an additional inherent advantage with a possibility of patterning directly on glass substrates. This renders these easily integrateable within miniaturized micro or optofluidic lab‐on‐a‐chip devices for rapid molecular diagnostics. We have thus, assembled an experimental EHSML set‐up consisting of a glass substrate as the bottom electrode on which a nano‐polymer film was deposited with ITO glass acting as a top electrode. Here, the EHL lithography was carried out under an inverted optical microscope, allowing an in situ observation of the pattern formation (Figure 5a and Movie S1, Supporting Information). In the EHSML directly patterned on glass, the generated micro‐ and nanopillars provide an inherent added value for improved, rapid biofluid separation and enhanced delivery to the SERS detection areas. This lays the platform for the integrated disposable lab‐on‐a‐chip architectures, which could include the SMART substrate together with a microfluidic processing cartridge, where biofluid is delivered across the sensor using capillary forces with the chip integrating simple, passive micro‐ and nanopumps (pillars) for a constant flowrate.^[^ ^75^ ^,^ ^76^ ^]^ In turn, this will yield the development of advanced micro‐optofluidic systems for rapid and high‐throughput separation of blood with built‐in sample preparation and detection, in a single‐step as well as further functional substrates transferred to a variety of systems, where continuous gradients will provide novel physical insights and functionalities.^[^ ^77^ ^]^ Overall, the fabricated SMART substrates demonstrated the ability to detect biomarkers within the TBI simulated plasma samples down to LoDs of 0.507 ± 0.033 pg mL^−1^ and EFs of (6.30 ± 0.12) × 10^9^ When combined with the computational SkiNET algorithm, these distinguished each glycan‐biomarker from a complex bio‐mixture. The presence of the imprinted specific binding sites allowed the preferential binding of the TBI biomarker at the optimized micronano‐pillars thus, significantly enhancing the SERS signal. Increased sensitivity and selectivity established for clinically significant biomarker LoDs, comparable to those present in the early stages of TBI, renders these substrates suitable for the detection of target biomarkers down to pM levels, laying the platform toward the detection at physiological concentrations. Furthermore, integration of the developed EHSML and the SMART substrates could be the used for detection of the TBI‐indicative glycans from blood plasma, CSF and other biofluids. These would enable direct detection of neuromarkers at the point‐of‐care (targeting <pg mL^−1^ range) due to their continuous efflux from neurons and build‐up of temporal profiles of extracellular activity during the various phases of brain injury. These would be subsequently, rapidly classified to distinguish between TBI patients and control cohorts, leveraging the excellent intrinsic sensing properties of advanced optofluidic lab‐on‐a‐chip platforms toward disruptive, real‐time diagnostic platform technologies.

## 3 Conclusions

Herein, through the rational design via hybrid EHL and synthetic nanochemistry delivering ultra‐selective plasmonic nanoroughness, down to picomolar level sensors for rapid molecular detection have been demonstrated. Micronano‐engineered tailor‐designed SMART substrates, with fine‐tuned architectures as individual detection centres for multiplexed measurements, deliver high sensitivity with increased selectivity, tuneability and timeliness, laying the platform toward advancing the field of point‐of‐care technologies for diagnostics and monitoring, particularly for neurological trauma. EHSML postures prominent advantages over other lithographic methods, including the inherent tuneability of the fabricated nano‐architectures through directly adjusting the various experimental parameters in a single‐step structuring, the speed of which can be easily controlled via the chosen material, that is, viscosity, to be patterned in the engineered high‐precision piezoelectric micro‐lithographic capacitor in conjunction with the designed and tailor‐fabricated dedicated top electrode. The tuneability of the EHSML enables multiple color‐banded active regions on a single substrate, with each hierarchical structure acting as an individual detection hub, matching the localized surface plasmon resonances to the excitation lasers to gain the highest enhancements combined with multiplex detection. The wavelengths of the localized surface plasmon resonances and the strength of the electric field are highly sensitive to the shape, dimensions and coupling modes of micro‐nanostructures, and therefore the fabricated SERS substrates with maximum enhancement at the desired excitation laser can be obtained via rational design for signals to occur at Stokes red‐shifted wavelengths. Such anisotropic localization of plasmons and SERS signal yields sensitive sensing down to the single molecular level.

Simultaneous multiplex detection is further established by resorting to spatial modulation of the fabricated SMART substrates, with each SERS‐active array specifically molecularly imprinted for different target molecules, enabling to analyze a variety of substances from biofluids. Surface molecular functionalization, via interactions with recognition motif monomers and the subsequent target removal, yields unique synthetic receptors rendering the overall EHSML platforms as highly sensitive and selective, capable of recognizing specific target molecules and concurrently yielding consistent high signal enhancements for rapid sensing. Moreover, the tuneable micro‐ and nanostructured substrates are cost‐effective, made from low‐cost polymers coated with a thin gold nano‐layer as well as scalable, and importantly can be easily integrated within a micro or optofluidic lab‐on‐a‐chip, due to their direct patternability on glass with the micro‐ and nanopillars providing inherently improved biofluid separation, flowrate and yield.

The acquired spectroscopic data can be rapidly classified using our new artificial neural network algorithm as a decision‐making support tool, enabling quantitative determination of target molecules, for example, glycan biomarkers, which in the long‐term, with clinical input on the validated panels, will achieve more accurate determination of relevant features with high efficiency and accuracy for development of specific diagnostic tools. Ultimate integration of SMART substrates with emerging artificial intelligence techniques, such as SkiNET and portable detector, will further provide important easily interpretable therapeutic and management technologies. The performance utility of EHSML‐fabricated SMART substrates’ capability to rapidly detect TBI‐indicative neuromarkers from spiked plasma samples has been demonstrated, achieving limits of detection down to picomolar levels for multiple glycans. This proof‐of‐concept lays the platform for further possibilities of tracking minute concentrations of neuro‐biomarkers, individually or as a panel, from clinical samples using SMART lab‐on‐a‐chip at various time points post neurological injury (based on the validated biomarkers for different phases of TBI including acute, subacute and chronic), which in turn will enable successful developments of improved interventions and sensing technologies for timely diagnostics and easy monitoring of a range of major neurological diseases, and would help avoid long‐term brain deficits and morbidity.

## 4 Experimental Section

### Materials

Ethanol, toluene and methanol solvents were purchased from Sigma‐Aldrich. Melezitose was purchased from Acros Organics. The benzyl‐terminated 3,3′‐disulfanediylbis(N‐phenylpropanamide) and benzoboroxole‐terminated 3,3′‐disulfanediylbis(N‐(1‐hydroxy‐1,3‐dihydrobenzo[c] oxaborol‐6‐yl) propanamide) were synthesized, as previously reported.^[^ ^78^ ^]^ The acrylamide‐terminated *N, N*′‐bis(acryloyl)cystamine, raffinose, stachyose and the GalNAc were purchased from Sigma‐Aldrich. A gold target with purity of 99.999% was purchased from Kurt J. Lesker. Polystyrene with a molecular weight of 100 kg mol^−1^ was used as the main polymer for the electrohydrodynamic lithography (Polymer Standards) and toluene was used as the main solvent in this process (Fisher Scientific). Highly polished *p*‐doped silicon wafers with <100> crystal orientation (Wafernet) were used during the EHL and the EHSML as bottom and top electrodes (X‐lith eXtreme Lithography). Materials were used as received with no further purification steps.

### Numerical Modelling

A 2D model of COMSOL Multiphysics v.4.3 with a commercial finite‐element model solver, was constructed to enable parametric studies. The model with the Floquet periodicity boundary conditions was applied to the system. The thickness of the gold layer covering the pillars was 18–23 nm. A *p*‐polarized plane wave, travelling normal to the surface, was used to illuminate the structures. The refractive index of the polymer and the surrounding medium was set at 1.3 and 1, respectively. The total width and depth of the model were 500 and 800 nm, respectively. Perfectly matched layers with a thickness of 12–50 nm were used to absorb the scattered radiation in all directions. The field intensity was taken either in the middle of the pillars or 1 nm away from them. In both cases, two points were examined, a point at the height of the pillars and a point halfway up the pillars. The electric field was calculated as a ratio of its value at a given point (1 nm away from the pillar edge) divided by the incident field that is, │*E*│/│*E*_0_│. SERS enhancement was calculated for the excitation wavelength and the measured Raman wavelength: (│*E*│_Excitation_/│*E*_0_│)^2^ × (│*E*│_Raman_/│*E*_0_│)^2^ The incident field of wavelengths at 633 nm propagates from the top in the (−z) direction with linear polarization in the x direction. The incident field amplitude was *E*_0_ = 1 V m^−1^. The model was solved for the scattered field of the micro nanostructures in a vacuum.

### EHSML Substrates Fabrication

Prior to the deposition of thin polymer film, the Si substrates were pre‐cut to 1 × 1 cm^2^ areas and thoroughly cleaned via double‐step procedure which commenced with the “piranha” solution consisting of 3:1 (98%) sulfuric acid to (30%) hydrogen peroxide, followed by rinsing with deionized water and drying under nitrogen flow and finally, subjected to a snow‐jet cleaning. Polymer nano films were subsequently deposited onto the cleaned silicon substrates. The top electrode of the micro‐capacitor was surface grafted with an octadecyl‐trichlorosilane self‐assembled monolayer to enable an easy mechanical to release of the substrate at the end of the lithographic process. The EHL rig was annealed above the glass transition temperature of the polymer whilst the micrometre was used to enable a rough adjustment of the inter‐electrode distance. Subsequently, a fine adjustment of the gap between the electrodes was achieved using the piezo, with the in‐built voltammeter sensor for in situ measuring of the current drawn by the rig during the patterning process. This yielded electrohydrodynamic instabilities, which resulted in patterning of micro‐nanostructures spanning the capacitor gap with controllable dimensions. For the EHSML patterning of glass substrates, ITO covered glass slides were used as substrates and the patterning was performed on top of an inverted optical microscope (Olympus GX61). The experiment commenced by either annealing the thin polymer film above glass transition temperature or introducing a saturated toluene atmosphere into the chamber, inducing swelling of the polymer film, while the capacitor plates were electrically grounded. The EHL structure formation was monitored via the transparent ITO covered glass substrates and recorded using the inverted optical microscope, connected computer throughout the experiment. After removal of the top plate, the quenched polymer film was further characterized by optical microscopy (Olympus BX60) and atomic force microscopy (AFM, Veeco Dimension 3100). The patterned substrates were coated with a thin nanogold layer (Emitech sputter‐coater) using a DC argon plasma and a gold target with purity of 99.999% (Kurt J. Lesker) via two subsequent cycles at 10 mA. Deposited benzenethiol (Analytical Standard, Sigma‐Aldrich) monolayer from ethanol solution was used as standard SERS analyte. Surface molecular functionalization was based on the processes described in refs. [42, 78] with gold‐coated patterned substrates initially, functionalized individually with benzyl‐terminated SAM and benzoboroxole‐terminated SAM, where 1 mm solution of benzyl‐terminated and benzoboroxole‐terminated self‐assembled monolayers in methanol were prepared. The subsequent creation of stable and high‐order complexes between oligosaccharides and benzoboroxoles was to be achieved by using optimal complexation conditions of 24 h stirring the mixture of glycans with an excess of 2‐(hydroxymethyl) phenylboronic acid cyclic monoester (8.0 equivalents per unit) and oligosaccharide in 6:1 (v/v) dioxane:acetonitrile at 90 °C with molecular sieves (3 Å) to remove water formed during the condensation reaction producing the complex. On the removal of the target oligosaccharide, a unique print remains surrounded by DSA‐oligosaccharide complex forming a specific binding pocket. Binding scaffolds to determine the selectivity to the target and not to non‐target oligosaccharides were achieved by immersing clean gold substrates in a 0.1 mm ethanolic solution of *N,N*′–bis(acryloyl)cystamine with 2% trifluoracetic acid for 24 h, as previously reported.

### Calculation of the SERS EF

SERS enhancement factor was calculated by comparing the intensities of the unenhanced Raman scattering, I_Raman_ peak at 1070 cm^−1^ of pure benzenethiol liquid obtained by focusing the laser into a quartz cell and the corresponding SERS signals, ISERS obtained from the SERS substrates. The detection volume of the solution‐phase benzenethiol sample, V_f_ was calculated using the following relation: V_f_ = (depth of focus) × (focus area) = (1.4 nλ/NA^2^) × π(0.4 λ/2NA).^2^ The surface density of the adsorbed benzenethiol molecules on the structured surface was taken as ρ_s_ = 3.3 molecules nm^−2^ and the enhanced area, A was defined as the diffraction limited spot size (= π(0.4 λ/2 NA)^2^). The enhancement factor was therefore, calculated using the relation: EF = [I_SERS_/(ρ_s_A)]/[I_Raman_/(ρ_v_V_f_).

### Scanning Electron and Atomic Force Microscopy Characterisation

The scanning electron microscopy (SEM) measurements were performed using a LEO ULTRA 55 SEM including a Schottky emitter (ZrO/W cathode) at acceleration voltages of 1–5 KV with a lateral resolution of 2–5 nm. Atomic force microscopy (AFM) measurements were performed using JPK NanoWizard II atomic force microscope was used to characterize the surfaces’ height, roughness and grain size and film thickness. The AFM measurements were performed using tapping mode via an intermittent contact mode of the cantilever tip with the sample in ambient conditions. NCHV‐A cantilevers with a resonance frequency of 320 kHz and stiffness of 42 Nm^−1^ were used. Height, roughness, grain size, and phase images were analyzed with Gwyddion's software (Version 2.55).

### SERS Measurements

SERS measurements were carried out with an *InVia^TM^* Confocal Raman Microscope System (Renishaw) equipped with 633 nm laser. The spectra were typically acquired with a 10 s exposure time and a laser power of 0.3–9 mW at the sample at 633 nm. SERS maps were acquired in Streamline mode (line scan) with 5 s of exposure time per pixel and a power of 6 mW. A 100× objective with a numerical aperture of 0.85 and a 50× objective with a 0.75 numerical aperture were used for measurements over a range of 500–2500 cm^−1^ relative to the excitation wavelength. When calculating the characteristic peak intensity obtained at different spectra, a consistent signal intensity for each target was observed. Polarization sensitive SERS was performed to confirm that the biomarkers retain natural state. An intelligent‐fitting filter was applied for baseline subtraction. Data analysis from the acquired spectra was performed using Python‐written algorithms. The peak intensities for prominent peaks throughout this work were gathered and later analyzed from the raw data following the baseline subtraction. Simulated SERS spectra were obtained through density functional theory calculations conducted with the ORCA 5.0.2 package.1 B3LYP with a def2‐SVP basis set selected and ran in parallel over CPU 28‐cores (AMD TR Pro 3975WX). Spectra were exported between 600–1800 cm^−1^ over 1024 points with 15 cm^−1^ peak width. The main assignments of the detected bands are based on refs. [65, 66, 67, 68].

### Data Classification and Statistical Analysis

Multi‐variate analysis was performed using the self‐optimizing Kohonen index network (SKiNET) artificial neural network algorithm.^[^ ^51^, ^79^ ^]^ SKiNET was based on the separation of data classes in a self‐organizing map (SOM) and an the undefining characterization using the self‐organizing map discriminant index (SOMDI), which appends a set of label vectors to each neuron and allows to study the most prominent features that cause the activation of a particular neuron to a class label, enabling the rapid subsequent classification of the tested data. SOM defines 2D maps of neurons, typically arranged as a grid of hexagons. Each neuron was assigned a weight vector, which is initialized randomly and had a length equal to the number of variables in a spectrum. The weight vector affects which neuron was activated for a given sample with the neighboring neurons having similar weights. Spatial clustering was then observed in the trained SOM, with spectra that exhibit distinct properties activating different neurons. To extract the information on which spectral features were responsible for certain neurons activating over others, SOMDI was used, providing a representation of weights associated with neurons identifying a particular class by introducing class vectors as labels for each spectrum and corresponding weight vectors for each neuron, without influencing the training process, allowing the identification of what type of data a given neuron activates, used to inspect the weights across all neurons and extract prominent features belonging to each class. Hexagons were coloured according to the modal class they activate, from the Raman spectra and those that have no majority class or activate none of the data were coloured white. For each class, there was a clearly defined block of neurons, with many of these activating only a single biomarker type and a higher SOMDI intensity indicates a greater importance of wavenumber. Further, by inputting a test sample into the trained neural network and detecting which neuron has been activated, the associated SOMDI provides class data, which was then used to make a prediction for the unseen sample, enabling SKiNET to be used a classifier. Raw component spectra from specific glycans were fitted to SOMDI for a particular state, constituting a physically realistic fit, as SERS spectra represented a mixed state of positive contributions from constituent components. The change in fitting coefficients were used to interpret the compositional changes to glycan biomarker in response to TBI. Comparisons across groups were performed by analysis of variance (ANOVA) and on transformed data. *p* < 0.05 was considered as significant. All analyses were carried out in FSPSS v.22 (IBM). For all the measurements, 95% confidence intervals were calculated. Statistical significance between two sites was tested using a two‐sided Student's *t*‐test.

### Receiver Operator Curve and Box Plots

ROC curves were generated for different cut‐off points using non‐parametric Mann–Whitney U and Kruskal‐Wallis tests, run using SPSS47. Each point in the ROC curve represented a sensitivity–specificity pair corresponding to a particular decision threshold and the values of sensitivity, specificity and accuracy were calculated using standard equations. A test with perfect discrimination (no overlap in the two distributions) had an ROC curve that passed through the upper left corner (100% sensitivity and 100% specificity). Therefore, the closer the ROC curve was to the upper left corner, the higher was the overall accuracy of the test. Box plots were generated using Vertex42 software, in which each series is an x‐y chart used to represent the quartiles and allows the data to include negative values. Classification sensitivity, accuracy and specificity were determined on the basis of: sensitivity = (TP)/(TP + FN), specificity = (TN)/(TN + FP) and accuracy = (TP + TN)/(TP + TN + FN + FP) with TP being “true positive,” TN “true negative,” FP “false positive,” and FN “false negative.” Association of each glycan biomarker signature with differentiation outcome (TBI versus HV) was evaluated using multiple statistical methods. The mean scores for glycan identification in the two groups were compared with a Wilcoxon rank‐sum test. *p*‐values < 0.05 were considered significant. The predictive value of each marker (GalNAc, Fuc6GlcNAc and Manα3) was first explored individually with logistic regression, and then the additive predicted value of the GalNAc/Fuc6GlcNAc/Manα3signature and the extent to which they interacted with each other was explored with a backward selection model. The final discrimination of the markers was identified with sensitivity‐selectivity analysis and the area under the curve (AUC) value of the was determined for receiver operator characteristic curves.

### Surface Plasmon Resonance Analysis

The capacity and affinity of the surfaces toward its respective target biomarker and non‐target biomarkers was determined using surface plasmon resonance system with a wide range of high‐quality molecular interaction data analysis (Biacore T200) at 25 °C using a running buffer. Prior to the affinity binding studies, a baseline was established by running degassed phosphate‐buffered saline through the surface plasmon resonance. For binding analysis, serially diluted gold glycans in phosphate‐buffered saline, as described previously^[^ ^80^ ^]^ were injected over coated surface molecularly functionalized substrates at a flow rate of 20 µL min^−1^, with the corresponding control glycans injected first in the reverse binding experiments with the dissociation initiated via flowing PBS over the substrates. The sensorgrams were analyzed by nonlinear curve fitting using a 1:1 interaction model or steady‐state affinity analysis using BIA evaluation software v.4.1 (GE Healthcare). Sensograms corresponding to the differences between the binding to the immobilized glycan and the binding to a blank well were used for analysis and were further referenced by subtracting appropriate control curves obtained by injecting the buffer alone.

### Principal Component Analysis

The minute differences in spectra were interpreted using a multivariate analysis which included the cluster separations of the different stages. PCA analyses was used with the Euclidean distance to infer how the separated clusters separated at each stage. The Euclidean distance between different cluster centroids was initially calculated by fitting an ellipsoid to the cluster. Subsequently, the ellipsoid centre position of principal components (PC1) and (PC2) was identified and used to calculate the Euclidean distance between clusters as: *d*_1→2_ = $\sqrt{{\left(PC1-PC1^{\prime }\right)}^{2}+{\left(PC2-PC2^{\prime }\right)}^{2}}$. PC and PC′ correspond to the different cluster centres and the distance error is considered proportional to the ellipse's major and minor axes. The cluster distance was compared for the varying molecular surface functionalization. PCA loadings are calculated based on the eigenvectors and eigenvalues obtained from the matrix operations to find the principal components (PC). The loadings have specific information related to SERS data, and depending on the PC space, the loadings exhibit different features. In a 2D PCA system, the different PC quadrants’ loadings are expressed as PC1 > 0 and PC2 > 0, PC1 < 0 and PC2 > 0, PC1 < 0 and PC2 < 0 and PC1 > 0 and PC2 < 0 with four different loading fingerprints related to the four quadrants.

### Spike and Recovery

The biomarkers were measured in blood plasma of healthy patients (*n* = 33) collected as part of the Golden Hour and ReCoS studies (Ethics refs.13/WA/0399 and11‐0429AP28). Participants were recruited through the Surgical Reconstruction and Microbiology Research Centre at Queen Elizabeth Hospital of Birmingham (UK). Written informed consents were received from participants or valid proxy (family or a professional not directly involved in the study) before inclusion. The study was approved by the National Research Ethics Service (IRAS ID 125 988). Samples were assessed by adding 50 µL of plasma sample and 10 µL of spike stock glycan marker solution to yield 0–50 pg mL^−1^ spike concentrations. Values for spiked samples reflected subtraction of the endogenous (no‐spike) value. Recovery for spiked test samples was calculated by comparison with the measured recovery of spiked diluent control. Diluent for the diluent control and preparation of spike stock solutions was the same as the standard diluent. All values represent the average of three replicates. Recovery was calculated as an (amount of compound found in spiked sample – amount of compound in sample)/amount of compound added × 100%. Once the recovery observed for the spike was nearly identical to the recovery obtained for the analyte prepared in standard diluent, the samples were considered as valid for the assay system.

### Determination of Limit of Detection and Limit of Quantification

LoDs were calculated based on the standard deviation of the response of the curve and the slope of the calibration curve at levels approximating the LoD according to the formula: LoD = 3.3(*σ* S^−1^). The standard deviation of the response was determined based on the standard deviation of *y*‐intercepts of regression lines. The calculation of the limit of quantification was based on the standard deviation of the response (*σ*) and the slope of the calibration curve (S) according to the formula: LoQ = 10(*σ* S^−1^).

## Conflict of Interest

The authors declare no conflict of interest.

## Supporting information

Supporting Information

Supplemental Movie 1

## Data Availability

The data that support the findings of this study are available from the corresponding author upon reasonable request.

## References

[1] K. W. Kho , K. Z. M. Qing , Z. X. Shen , I. B. Ahmad , S. S. C. Lim , S. Mhaisalkar , T. J. White , F. Watt , K. C. Soo , M. Olivo , J. Biomed. Opt. 2008, 13, 054026. 19021406 10.1117/1.2976140

[2] C. Lim , J. Hong , B. G. Chung , A. J. Demello , J. Choo , Analyst. 2010, 135, 837. 20419230 10.1039/b919584j

[3] S. Pennathur , D. K. Fygenson , Lab Chip. 2008, 8, 649. 18432330 10.1039/b805064n

[4] T. J. Moore , A. S. Moody , T. D. Payne , G. M. Sarabai , A. R. Daniel , B. Sharma , Biosensors (Basel). 2018, 8, 46. 29751641 10.3390/bios8020046 PMC6022968

[5] T. Itoh , A. Sujith , Y. Ozaki , in Frontiers of Molecular Spectroscopy, (Ed. J. Laane ), Elsevier, Amsterdam 2009.

[6] S. Schlücker , W. Kiefer , in Frontiers of Molecular Spectroscopy, (Ed. J. Laane ), Elsevier, Amsterdam 2009.

[7] H. Ma , X. Tang , Y. Liu , X. X. Han , C. He , H. Lu , B. Zhao , Anal. Chem. 2019, 91, 8767. 31251021 10.1021/acs.analchem.9b01956

[8] Y. Wang , S. Kang , A. Khan , G. Ruttner , S. Y. Leigh , M. Murray , S. Abeytunge , G. Peterson , M. Rajadhyaksha , S. Dintzis , S. Javid , J. T. C. Liu , Sci. Rep. 2016, 6, 21242. 26878888 10.1038/srep21242 PMC4754709

[9] Y. W. Wang , A. Khan , M. Som , D. Wang , Y. Chen , S. Y. Leigh , D. Meza , P. Z. McVeigh , B. C. Wilson , J. T. C. Liu , Technology. 2014, 2, 118. 25045721 10.1142/S2339547814500125 PMC4103661

[10] R. Mcqueenie , R. Stevenson , R. Benson , N. Macritchie , I. Mcinnes , P. Maffia , K. Faulds , D. Graham , J. Brewer , P. Garside , Anal. Chem. 2012, 84, 5968. 22816780 10.1021/ac3006445

[11] W. L. Barnes , A. Dereux , T. W. Ebbesen , Nature. 2003, 424, 824. 12917696 10.1038/nature01937

[12] K. Kneipp , Y. Wang , H. Kneipp , L. T. Perelman , I. Itzkan , R. R. Dasari , M. S. Feld , Phys. Rev. Lett. 1997, 78, 1667. 10.1103/PhysRevLett.76.2444 10060701

[13] S. Nie , S. R. Emory , Science. 1997, 275, 1102. 9027306 10.1126/science.275.5303.1102

[14] E. Ozbay , Science. 2006, 311, 189. 16410515 10.1126/science.1114849

[15] U. S. Dinish , G. Balasundaram , Y. T. Chang , M. Olivo , J Biophotonics. 2014, 7, 956. 23963680 10.1002/jbio.201300084

[16] U. S. Dinish , G. Balasundaram , Y.‐T. Chang , M. Olivo , Sci. Rep. 2014, 4, 4075. 24518045 10.1038/srep04075 PMC3921631

[17] N. D. Israelsen , C. Hanson , E. Vargis , ScientificWorldJournal. 2015, 2015, 124582. 25884017 10.1155/2015/124582 PMC4390178

[18] R. Tantra , R. J. C. Brown , M. J. T. Milton , J. Raman Spectrosc. 2007, 38, 1469.

[19] B. D. Piorek , S. J. Lee , J. G. Santiago , M. Moskovits , S. Banerjee , C. D. Meinhart , Proc. Natl. Acad. Sci. USA. 2007, 104, 18898. 18025462 10.1073/pnas.0708596104 PMC2141879

[20] X. Qian , X. Zhou , S. Nie , J. Am. Chem. Soc. 2008, 130, 14934. 18937463 10.1021/ja8062502 PMC3729406

[21] M. Kahl , E. Voges , S. Kostrewa , C. Viets , W. Hill , Sens. Actuators, B. 1998, 51, 285.

[22] S. Bhalla , D. T. Melnekoff , A. Aleman , V. Leshchenko , P. Restrepo , J. Keats , K. Onel , J. R. Sawyer , D. Madduri , J. Richter , S. Richard , A. Chari , H. J. Cho , J. T. Dudley , S. Jagannath , A. Laganà , S. Parekh , Sci. Adv. 2021, 7, eabg9551. 34788103 10.1126/sciadv.abg9551 PMC8598000

[23] S. Y. Chou , P. R. Krauss , P. J. Renstrom , Science. 1996, 272, 85.

[24] B. Yan , A. Thubagere , W. R. Premasiri , L. D. Ziegler , L. Dal Negro , B. M. Reinhard , ACS Nano. 2009, 3, 1190. 19354266 10.1021/nn800836f

[25] N. L. Garrett , P. Vukusic , F. Ogrin , E. Sirotkin , C. P. Winlove , J. Moger , J. Biophotonics. 2009, 2, 157. 19343696 10.1002/jbio.200810057

[26] D. R. Davies , G. H. Cohen , Proc. Natl. Acad. Sci. USA 1996, 93, 7.8552677

[27] J. De Gelder , K. De Gussem , P. Vandenabeele , L. Moens , J. Raman Spectrosc. 2007, 38, 1133.

[28] B. Friguet , A. F. Chaffotte , L. Djavadi‐Ohaniance , M. E. Goldberg , J. Immunol. Methods. 1985, 77, 305. 3981007 10.1016/0022-1759(85)90044-4

[29] E. J. Sundberg , R. A. Mariuzza , Adv. Protein Chem. 2002, 61, 119. 12461823 10.1016/s0065-3233(02)61004-6

[30] Z. Wang , S. Zong , L. Wu , D. Zhu , Y. Cui , Chem. Rev. 2017, 117, 7910. 28534612 10.1021/acs.chemrev.7b00027

[31] R. S. Yalow , S. A. Berson , Nature. 1959, 184, 1648. 13846363 10.1038/1841648b0

[32] H. Cho , B. R. Baker , S. Wachsmann‐Hogiu , C. V. Pagba , T. A. Laurence , S. M. Lane , L. P. Lee , J. B.‐H. Tok , Nano Lett. 2008, 8, 4386. 19367849 10.1021/nl802245w PMC3477626

[33] J. Hu , P.‐C. Zheng , J.‐H. Jiang , G.‐L. Shen , R.‐Q. Yu , G.‐K. Liu , Anal. Chem. 2009, 81, 87. 19117446 10.1021/ac801431m

[34] K. Carron , L. Peitersen , M. Lewis , Environ. Sci. Technol. 1992, 26, 1950.

[35] L. G. Crane , D. Wang , L. M. Sears , B. Heyns , K. Carron , Anal. Chem. 1995, 67, 360.

[36] A. Pal , D. L. Stokes , J. P. Alarie , T. Vo‐Dinh , Anal. Chem. 1995, 67, 3154.

[37] T. Vo‐Dinh , D. L. Stokes , Field Anal. Chem. Technol. 1999, 3, 346.

[38] K. Kantarovich , I. Tsarfati , L. A. Gheber , K. Haupt , I. Bar , Anal. Chem. 2009, 81, 5686. 19601651 10.1021/ac900418x

[39] Y. Lv , Y. Qin , F. Svec , T. Tan , Biosens. Bioelectron. 2016, 80, 433. 26874111 10.1016/j.bios.2016.01.092

[40] J. Langer , I. García , L. M. Liz‐Marzán , Faraday Discuss. 2017, 205, 363. 28880321 10.1039/c7fd00123a

[41] D. Lin , G. Cohen Freue , Z. Hollander , G. B. John Mancini , M. Sasaki , A. Mui , J. Wilson‐Mcmanus , A. Ignaszewski , C. Imai , A. Meredith , R. Balshaw , R. T. Ng , P. A. Keown , W. Robert Mcmaster , R. Carere , J. G. Webb , B. M. Mcmanus , J. Heart Lung Transplant. 2013, 32, 723. 23796154 10.1016/j.healun.2013.04.011

[42] A. Stephenson‐Brown , A. L. Acton , J. A. Preece , J. S. Fossey , P. M. Mendes , Chem. Sci. 2015, 6, 5114. 29142730 10.1039/c5sc02031j PMC5666680

[43] R. Xing , Y. Ma , Y. Wang , Y. Wen , Z. Liu , Chem. Sci. 2019, 10, 1831. 30842851 10.1039/c8sc04169e PMC6369433

[44] F. Wang , S. Cao , R. Yan , Z. Wang , D. Wang , H. Yang , Sensors (Basel). 2017, 17, 2689. 29160798 10.3390/s17112689 PMC5713634

[45] L. Chen , X. Wang , W. Lu , X. Wu , J. Li , Chem. Soc. Rev. 2016, 45, 2137. 26936282 10.1039/c6cs00061d

[46] P. K. Dash , J. Zhao , G. Hergenroeder , A. N. Moore , Neurotherapeutics. 2010, 7, 100. 20129502 10.1016/j.nurt.2009.10.019 PMC5084117

[47] G. Reddy , S. Gopinath , C. Robertson , Semin. Neurol. 2016, 36, 570. 27907961 10.1055/s-0036-1592169

[48] S. T. Dekosky , M. D. Ikonomovic , S. Gandy , N. Engl. J. Med. 2010, 363, 1293. 20879875 10.1056/NEJMp1007051

[49] N. Carney , A. M. Totten , C. O'reilly , J. S. Ullman , G. W. J. Hawryluk , M. J. Bell , S. L. Bratton , R. Chesnut , O. A. Harris , N. Kissoon , A. M. Rubiano , L. Shutter , R. C. Tasker , M. S. Vavilala , J. Wilberger , D. W. Wright , J. Ghajar , Neurosurgery. 2017, 80, 6. 27654000 10.1227/NEU.0000000000001432

[50] I. Pozzato , S. Meares , A. Kifley , A. Craig , M. Gillett , K. V. Vu , A. Liang , I. Cameron , B. Gopinath , BMJ Open. 2020, 10, e034494. 10.1136/bmjopen-2019-034494 PMC7045153 32019818

[51] C. Banbury , R. Mason , I. Styles , N. Eisenstein , M. Clancy , A. Belli , A. Logan , P. Goldberg Oppenheimer , Sci. Rep. 2019, 9, 10812. 31346227 10.1038/s41598-019-47205-5 PMC6658481

[52] C. Busà , J. J. S. Rickard , E. Chun , Y. Chong , V. Navaratnam , P. Goldberg Oppenheimer , Nanoscale. 2017, 9, 1625. 28074956 10.1039/c6nr08706j PMC5433428

[53] P. Goldberg‐Oppenheimer , D. Eder , U. Steiner , Adv. Funct. Mater. 2011, 21, 1895.

[54] P. Goldberg‐Oppenheimer , T. Hutter , B. Chen , J. Robertson , S. Hofmann , S. Mahajan , J. Phys. Chem. Lett. 2012, 3, 3486. 26290977 10.1021/jz301333r

[55] J. J. S. Rickard , I. Farrer , P. Goldberg‐Oppenheimer , ACS Nano. 2016, 10, 3865. 26905779 10.1021/acsnano.6b01246 PMC4819533

[56] P. Goldberg‐Oppenheimer , U. Steiner , Small. 2010, 6, 1248. 20486223 10.1002/smll.201000060

[57] P. D. C. Gomes , J. J. S. Rickard , P. Goldberg Oppenheimer , ACS Appl. Nano Mater. 2020, 3, 6774. 32743351 10.1021/acsanm.0c01190 PMC7386576

[58] A. Pal , M. Bérubé , D. G. Hall , Angew. Chem., Int. Ed. 2010, 49, 1492. 10.1002/anie.200906620 20084659

[59] M. Kvist , L. Valimaa , A. Harel , J. P. Posti , M. Rahi , I. Saarenpaa , M. Visuri , A. Ostberg , J. Rinne , Brain Sci. 2021, 11, 1480. 34827479 10.3390/brainsci11111480 PMC8615782

[60] P. G. Oppenheimer , Electrohydrodynamic Patterning of Functional Materials, Springer, Cham 2013.

[61] S. Mahajan , R. M. Cole , B. F. Soares , S. H. Pelfrey , A. E. Russell , J. J. Baumberg , P. N. Bartlett , J. Phys. Chem. C. 2009, 113, 9284.

[62] J. Zhang , J. Li , S. Tang , Y. Fang , J. Wang , G. Huang , R. Liu , L. Zheng , X. Cui , Y. Mei , Sci. Rep. 2015, 5, 15012. 26443526 10.1038/srep15012 PMC4595732

[63] A. K. Singh , S. A. Khan , Z. Fan , T. Demeritte , D. Senapati , R. Kanchanapally , P. C. Ray , J. Am. Chem. Soc. 2012, 134, 8662. 22559168 10.1021/ja301921k

[64] P. De Carvalho Gomes , M. Hardy , Y. Tagger , J. J. S. Rickard , P. Mendes , P. G. Oppenheimer , J. Phys. Chem. C. 2022, 126, 13774. 10.1021/acs.jpcc.2c03524 PMC9393890 36017358

[65] G. Socrates , Infrared and Raman Characteristic Group Frequencies: Tables and Charts, Wiley, Chichester 2001.

[66] P. Larkin , Psychosocial sources of aggression in young adults with intellectual disabilities. PhD thesis, University of Glasgow, Scotland 2011.

[67] A. C. S. Talari , Z. Movasaghi , S. Rehman , I. R. Rehman , Appl. Spectrosc. Rev. 2015, 50, 46.

[68] C. Krafft , L. Neudert , T. Simat , R. Salzer , Spectrochim. Acta, Part A 2005, 61, 1529. 10.1016/j.saa.2004.11.017 15820887

[69] S. Mondello , V. Sandner , M. Goli , E. Czeiter , K. Amrein , P. M. Kochanek , S. Gautam , B. G. Cho , R. Morgan , A. Nehme , G. Fiumara , A. H. Eid , C. Barsa , M. A. Haidar , A. Buki , F. H. Kobeissy , Y. Mechref , EClinicalMedicine. 2022, 50, 101494. 35755600 10.1016/j.eclinm.2022.101494 PMC9218141

[70] S.‐U. Lee , A. Grigorian , J. Pawling , I.‐J. Chen , G. Gao , T. Mozaffar , C. Mckerlie , M. Demetriou , J. Biol. Chem. 2007, 282, 33725. 17855338 10.1074/jbc.M704839200

[71] A. U. Brandt , M. Sy , J. Bellmann‐Strobl , B. L. Newton , J. Pawling , H. G. Zimmermann , Z. Yu , C. Chien , J. Dörr , J. T. Wuerfel , J. W. Dennis , F. Paul , M. Demetriou , JAMA Neurol. 2021, 78, 842. 33970182 10.1001/jamaneurol.2021.1116 PMC8111565

[72] R. Kleene , M. Schachner , Nat. Rev. Neurosci. 2004, 5, 195. 14976519 10.1038/nrn1349

[73] C. Moenninghoff , O. Kraff , S. Maderwald , L. Umutlu , J. M. Theysohn , A. Ringelstein , K. H. Wrede , C. Deuschl , J. Altmeppen , M. E. Ladd , M. Forsting , H. H. Quick , M. Schlamann , PLoS One. 2015, 10, e0122329. 25793614 10.1371/journal.pone.0122329 PMC4368671

[74] M. Sy , A. U. Brandt , S.‐U. Lee , B. L. Newton , J. Pawling , A. Golzar , A. M. A. Rahman , Z. Yu , G. Cooper , M. Scheel , F. Paul , J. W. Dennis , M. Demetriou , J. Biol. Chem. 2020, 295, 17413. 33453988 10.1074/jbc.RA120.015595 PMC7762951

[75] N. Vasilakis , K. I. Papadimitriou , H. Morgan , T. Prodromakis , Microfluid. Nanofluid. 2017, 21, 103. 32025228 10.1007/s10404-017-1935-2 PMC6979692

[76] H. Madadi , J. Casals‐Terré , R. Castilla‐López , M. Sureda‐Anfres , Microfluid. Nanofluid. 2014, 17, 115.

[77] M. Schöttle , T. Lauster , L. J. Roemling , N. Vogel , M. Retsch , Adv. Mater. 2023, 35, 2208745. 10.1002/adma.202208745 36366915

[78] S. Tommasone , Y. K. Tagger , P. M. Mendes , Adv. Funct. Mater. 2020, 30, 2002298. 32774200 10.1002/adfm.202002298 PMC7405978

[79] E. Buchan , L. Kelleher , M. Clancy , J. J. Stanley Rickard , P. G. Oppenheimer , Anal. Chim. Acta. 2021, 339074, 1185. 10.1016/j.aca.2021.339074 34711319

[80] A. Varki , Glycobiology. 2017, 27, 3. 27558841 10.1093/glycob/cww086 PMC5884436

