# Supplementary material for: Advanced Tuneable Micronanoplatforms for Sensitive and Selective Multiplexed Spectroscopic Sensing via Electro‐Hydrodynamic Surface Molecular Lithography
Source: Adv Sci (Weinh). 2024 Jan 15;11(12):2306068. doi: 10.1002/advs.202306068 (PMC10966563; doi:10.1002/advs.202306068)
Supplement: Supplementary file 1 — Supporting Information [file ADVS-11-2306068-s001.pdf]

ADVANCED SCIENCE
Open Access

Supporting Information

for *Adv. Sci.*, DOI 10.1002/advs.202306068

Advanced Tuneable Micronanoplatforms for Sensitive and Selective Multiplexed Spectroscopic Sensing via Electro-Hydrodynamic Surface Molecular Lithography

*Paulo De Carvalho Gomes, Martin Hin-Chu, Jonathan James Stanley Rickard and Pola Goldberg Oppenheimer**

# - Supporting Information -

Advanced Tuneable Nanoplatforms for Sensitive and Selective Multiplexed Spectroscopic Sensing *via* Electro-Hydrodynamic Surface Molecular Lithography

Paulo De Carvalho Gomes, Matin Hin-Chu, Jonathan James Stanley Rickard and Pola Goldberg Oppenheimer

S1. Theoretical Background and Physical Principles of the EHL:

In the engineered micronano capacitor rig, the EHL process commenced with annealing a polymer film with initial thickness, h deposited onto a bottom silicon substrate, annealed above the glass transition temperature T_g, yielding a flow of the viscous Newtonian fluid. This leads to the development of a spectrum of capillary waves at the liquid surface due to the molecular motion of the fluid at finite temperatures where the capillary waves, as perturbations of the free interface, generate a hydrodynamic flow in the film. The evolution of such capillary wave spectrum, assuming a sinusoidal fluctuation with wave number $q = 2\pi/\lambda$ and amplitude ζ (with $\zeta \ll h$), is described by the dispersion relation,

$$h\,(x,\,t) - \;h = Re\,\{\zeta e^{iqx + t/\tau}\} \qquad [1]$$

which relates the time constant τ to q and establishes whether fluctuations with wavelength, λ (with $\lambda \gg h$) are exponentially amplified or suppressed. The modulation of the free interface determines liquid material transport in the plane of the film described by the Navier-Stokes equation, which yields the velocity profile, v in the nanofilm. This, with an equation of continuity for the system, establishes the hydrodynamics of the polymer-air interface in the EHL rig. Given that for an infinitesimal volume element moving with the fluid accelerated by the forces acting on it and the thickness of the film is much smaller than the lateral extent of the film where the flow of liquid primarily takes place in the lateral direction, yields the vector equation:

$$\rho(\partial v/\partial t) + \rho[u(\partial v/\partial x) + v(\partial v/\partial y) + w(\partial v/] = -\partial p/\partial x + \eta\,(\partial^2 v/\partial x^2 + \partial^2 v/\partial y^2 + \partial^2 v/\partial z^2) + \rho g \qquad [2]$$

where, the velocity components u, v, w are the dependent variables and g is the gravitational acceleration. To calculate the dominant wavelength of the EHL instabilities, Eq. [2] can be simplified. Since the high viscosity of polymer melt results in a low flow velocity, the quadratic terms can be neglected.

The resulting dynamics are slow, meaning that the velocity profile can be considered in a quasi-steady state and for thin films with dimensions smaller than the capillary constant, gravity does not have an influence on the shape of the interface. Additionally, the fluctuations have a small amplitude compared to the film thickness. Finally, for $\lambda \gg h$ and $\zeta \ll h$, the flow in the nano-film can be approximated as steady laminar flow caused by a pressure gradient in the lateral direction and the velocity gradient varies only along the z-axis. These considerations yield the simplified equation:

$$0 = -\partial p/\partial x + \eta(\partial^2 v/\partial z^2) \qquad [3]$$

with the mean velocity in the film of

$$v_{\mathrm{avg}} = 1/h \int v(z)\,dz = h2/3\eta(-\partial p/\partial x) \qquad [4]$$

describing a *Poiseuille*-type flow in the liquid film in the presence of a pressure gradient. From Eqs. [3]-[4], any change in the height of the thin polymer film results in a flow in the lateral direction redistributing the fluid from the valleys to the peaks of the undulations. To establish the dynamics of the liquid-air interface, the interfacial pressure acting at the surface separating the two media must be determined. The total interfacial pressure is comprised of several contributions including (i) the ambient air pressure, which is independent of the film thickness for h<capillary length, (ii) the van der Waals intermolecular forces, which act between all atoms and molecules, (iii) the Laplace pressure stemming from the curvature of the interface and (iv) any excess surface pressure, which is the electrostatic pressure induced by externally applied electric field, enabling the electrohydrodynamic pattern formation process.

During the EHL, due to the small inter-electrode distance, a strong electric field (on the order of 10^8V/m) is generated inside the micro-capacitor rig, meaning that the electrostatic forces are much stronger than the van der Waals pressure, which can be neglected. For a dielectric liquid polymer in a capacitor, the applied potential difference, V, across the two electrodes (at distance d) gives rise to a strong electric field, E_f across the dielectric material. The E_f causes the energetically unfavourable build-up of displacement charges at the dielectric interface, resulting in an effective surface charge density. The charges at the polymer-air interface experience an effective attraction with the oppositely charged upper electrode. The electric field thus aligns the interface separating the two media parallel to the field lines while minimising the free electrostatic energy of the system. The strong electrostatic pressure (on the order of 100 N/m^2) at the liquid-air interface subsequently overcomes the stabilising effects of the surface tension (<γ>=30 mN/m) and destabilises the nano-film:

$$p_{el} = -1/2\varepsilon_0\varepsilon_p(\varepsilon_p - 1)E_f^{\,2} \quad [5]$$

where, ε_p is the dielectric constant of the polymer (typically, 2.5) and ε_0 is the dielectric permittivity of the vacuum. The electric field in the polymer is:

$$E_f = V/\,[\varepsilon_p d - (\varepsilon_p - 1)\,h] \quad [6]$$

where, the exponentially fastest-growing mode, given by the maximum of the dispersion relation, dominates the mode spectrum.

$$\lambda_{max} = 2\pi\sqrt{-2\gamma/[\partial p_{el}/\,\partial \mathrm{h}]} = 2\pi\sqrt{\frac{\gamma V}{\varepsilon_0\varepsilon_p(\varepsilon_p-1)^2}E_f^{-\frac{3}{2}}} \quad [7]$$

In the capillary regime, the surface tension, γ (*i.e.,* the intrinsic stabilising Laplace Pressure) is the dominant restoring force which opposes the formation of surface fluctuations that increase the surface area. The generated strong E_f at the polymer-air interface overcomes the surface tension and couples to the capillary wave spectrum, amplifying surface instabilities with a characteristic wavelength, λ. This leads to a lateral redistribution of the film from surrounding thinning regions, further pinned to the top electrode and detached from the surrounding polymer film by draining the liquid bridge. Subsequently, resulting in the rearrangement to the energetically favourable configuration of the material, yielding the electrohydrodynamically patterned structures on the substrate.

These equations and the overall underpinning linear stability analysis of the EHL patterning process, it is evident that there is an inner-dependence of experimentally controllable parameters *e.g.,* initial film thickness, *h*, inter-electrode spacing, *d*, surface tension (from the Laplace pressure) as well as polymer viscosity η arising from the exponentially fastest growing mode, *i.e.,* $\lambda=2\pi/q_{max}$, where τ is a characteristic constant for the temporal evolution of mode q and is directly proportional to polymer's viscosity: $\tau=3\eta/\gamma h^3 q^4$.

These mean that by varying the experimental parameters of initial film thickness, inter-electrode spacing, surfaces tension and viscosity of the polymer used as well as the externally applied voltage (and hence E_f) allow to control the formed micro- and nanostructures. For instance, whilst varying the initial polymer film thickness along with the electrode gap will control the aspect ratio of the formed structures (*i.e.,* height to width variation), varying the strength of the E_f or the applied voltage, will control the dominant wavelength of the pattern evolution, which in turn, determines the pitch (*i.e.,* the gaps between) the formed structures. Changing the viscosity of the polymer in the capacitive gap, will dictate the speed of the patterning (*i.e.,* onset of the instability formation). Reducing for example the interfacial surface tension further accelerates pattern replication and reduces the characteristic pattern size.

Choice of the polymer on the other hand allows to control the applications of interest *e.g.,* using a more hydrophobic polymer enables additional micro- and nanostructure and generation of superhydrophobic surfaces, leaky dielectric

polymer allows structuring conductive substrates in a single step, integrated nanocomposite polymer matrices allowed structuring FET / LED like substrates *etc*. Importantly, in the lithographic EHL mode, electrostatically induced structure formation is not limited to the intrinsic wavelength, allowing the replication of a large range in feature sizes for otherwise identical experimental parameters.

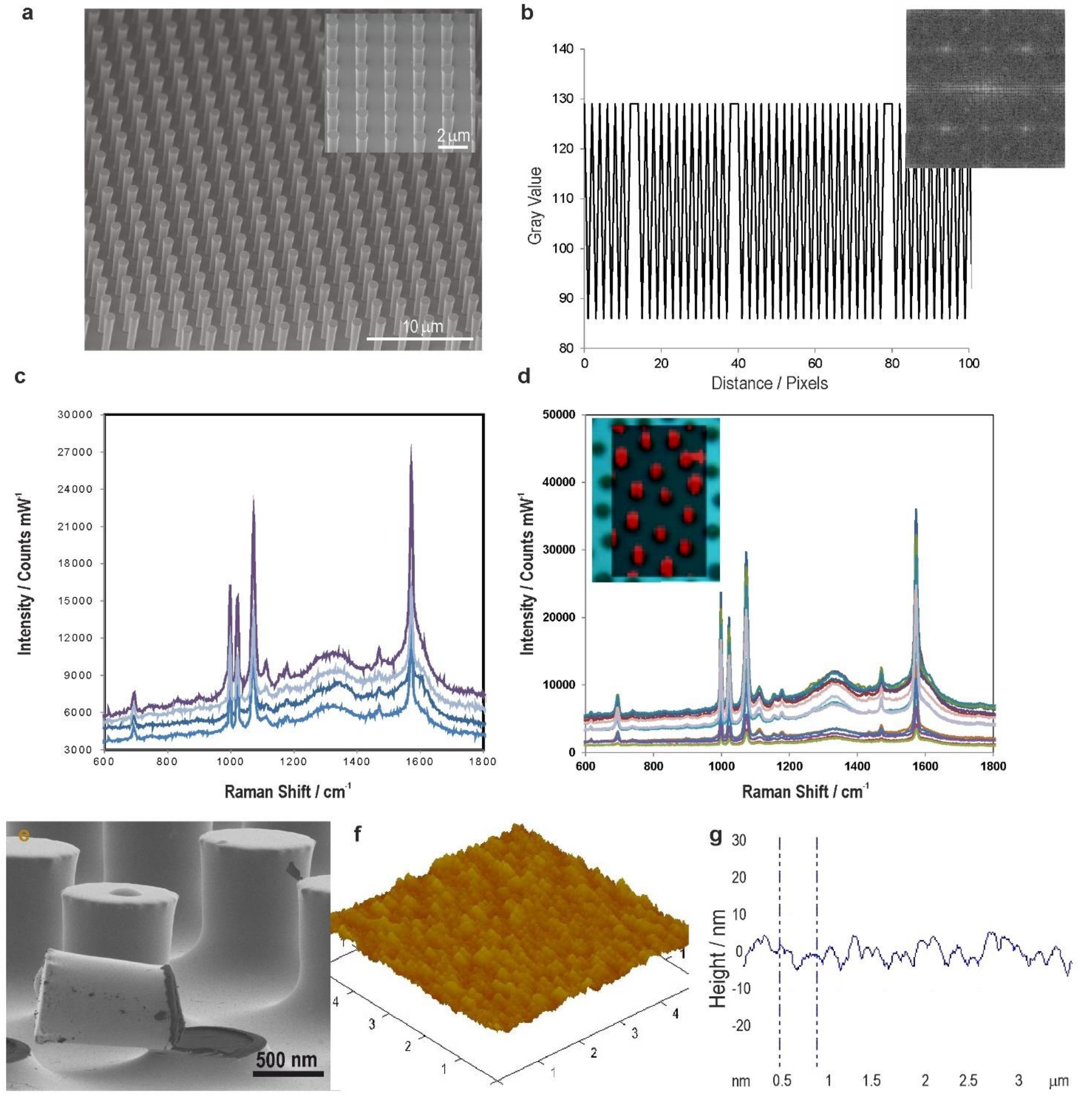

Figure S1. (a). SEM image of the EHL fabricated micronano-structured platforms with a high-level of uniformity across several indiscriminate areas on each substrate, ingrained by the plot profile **(b)** of the grey value distribution *versus* distance across the image, demonstrating consistent structural units across the entire area. **(Inset).** Corresponding fast Fourier transform image with low and high frequencies showing a similar image information with two dominating passing through the centre, both originating from the uniform structures in the background of the original image. **(c-d).** The homogenous substrates provide a consistent SERS signal over the large substrate surface area, yielding uniform signal. **(c).** SERS spectra acquired from six arbitrary locations across each substrate, from seventeen substrates **(d)** using benzenethiol monolayer as a standard analyte, yielded reproducible SERS intensities due to the optimised conformation of the underlying surfaces with variations of less than 9% in relative standard deviation (s.d.e.v.) and smaller than 3.9% in terms of the relative peak intensities. SERS intensity map **(d, inset)** of the characteristic benzenethiol 1070 cm^{-1} band overlaid on top of the top an optical microscopy image, demonstrating that substrates' uniform signal over the surface. **(e).** Low-angle backscattered SEM cross-section image of the Au-coated substrates confirming uniform and conformal coverage of the deposited nanolayer (brighter outer coating) following the pillars (dark internal core). **(f).** 3D AFM images of Au layer deposited *via* three successive sputtering cycles at 70 mA and a **(g)** representative cross-section of the AFM images of 12nm gold layer demonstrate that the overall average roughness after gold deposition is of the order of tens nanometres.

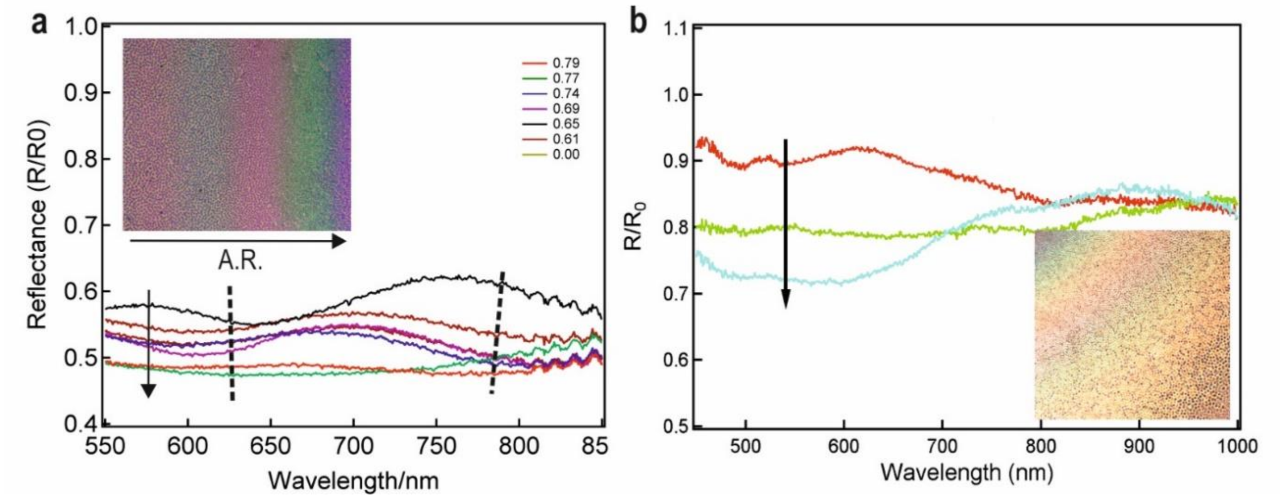

Supporting Figure S2. Tuneability of structural dimensions of the metallodielectric photonic EHSML generated SERS platforms was assessed for controllable plasmon excitations. **(a).** Spectrally resolved reflectance, *R* spectra (normalised to flat gold, R_0) across a sample with a range of aspect ratio of pillars, generating bands of colour corresponding to the various aspect ratios **(inset)** and **(b)** the variation of the extinction as function of gap between the pillars to their diameter at the excitation wavelength of 633nm (dashed lines) (*n*=3). The arrow corresponds to the variation in aspect ratios and to the colour change across the sample arising from this variation.

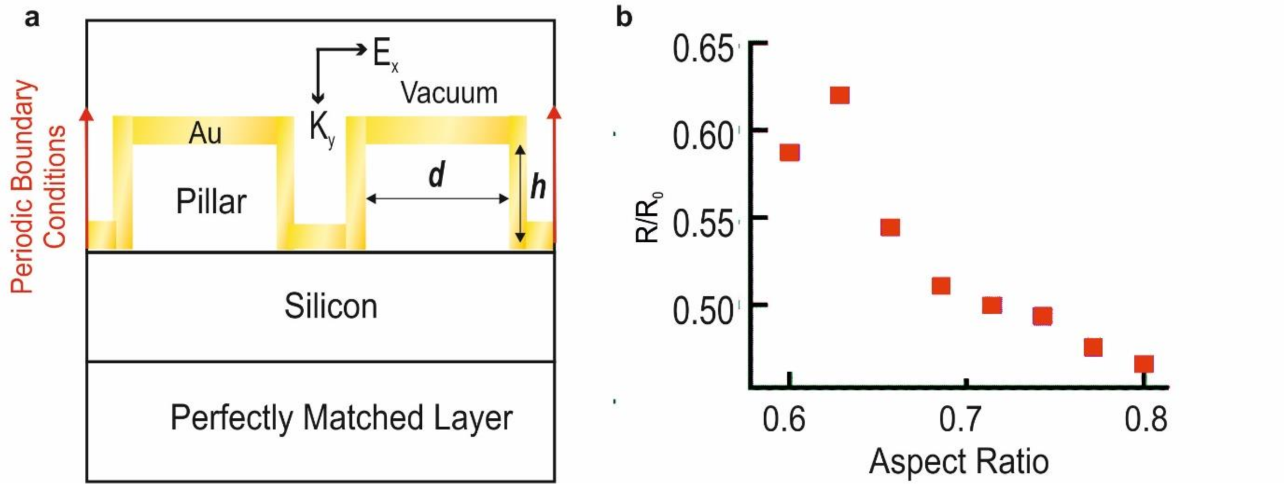

Figure S3. (a). To design and optimise SERS signal of the gold coated substrates for various aspect ratios, the scattered electric field intensity in vacuum was simulated using a finite model, with a plane-wave incident normal to the substrate and a linear polarization perpendicular to the top edge. **(b).** Dependence of the field intensity with periodic boundary conditions reveals increasing intensity with an increasing aspect ratio up to optimal dimensions at *f*=0.81. Experimentally measured reflectance across pillars, R/R_0 with laterally varying aspect ratios exhibits increased extinction with increasing aspect ratio with a nearly 30% increase occurs in extinction when varying the aspect ratio from 0.6 to 0.8. This extinction primarily arises from coupling of light into plasmon resonances which are tuned by the pillar geometry. This shows that the relative SERS enhancement (**Fig. 1g, Figs. 3a-c**) is correlated with the increased strength of plasmonic coupling into the structure **(b)**. The tuneability of the aspect ratio and its effect on strength of plasmon resonances and SERS allows the optimization of substrates for different laser excitation wavelengths.

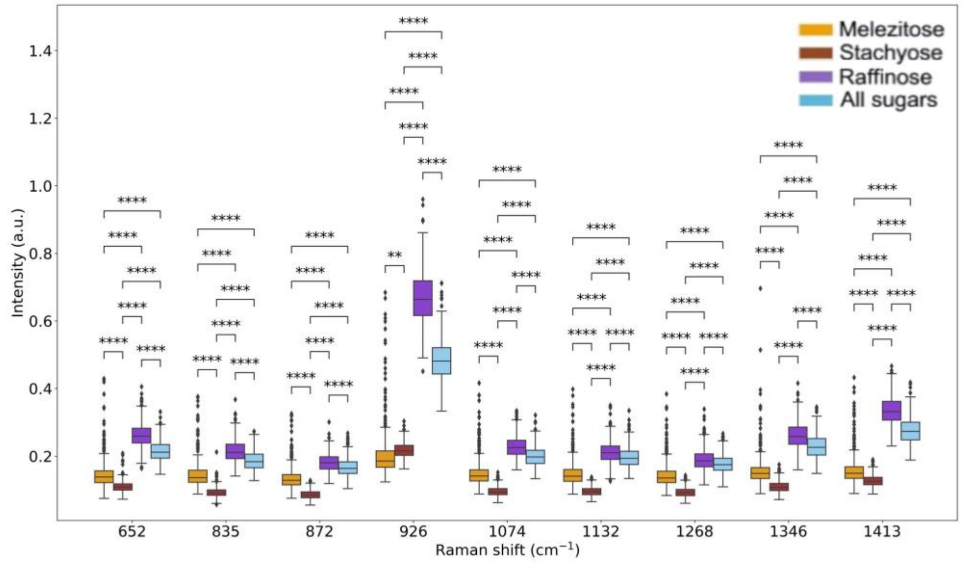

Figure S4. Normalised intensity box and whisker plots reveal statistically significant differentiation of the sugars, arising from the SERS intensity differences identified from the spectral peaks outlined by dashed lines in Fig. 4c.

a

Sample	No Spike (0pg/mL)	Spiked (50pg/mL)
Diluent	0	50.7
1	0	51.2
2	0.7	48
3	0	48.9
4	0	52.9
5	0	50.3
6	0.7	49.9
7	0	51.6
8	0.3	50.9
9	0.7	47.1
10	2.1	49
Average	0.409090909	50.04545455
**Recovery %**		**98.70898332**
**STDEV**		**1.685445719**

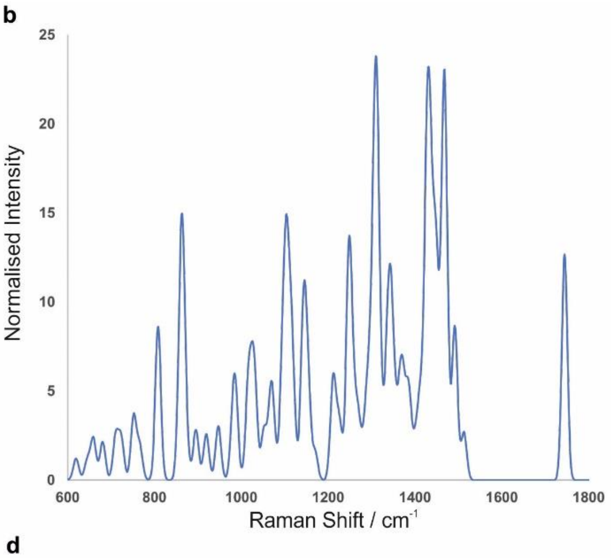

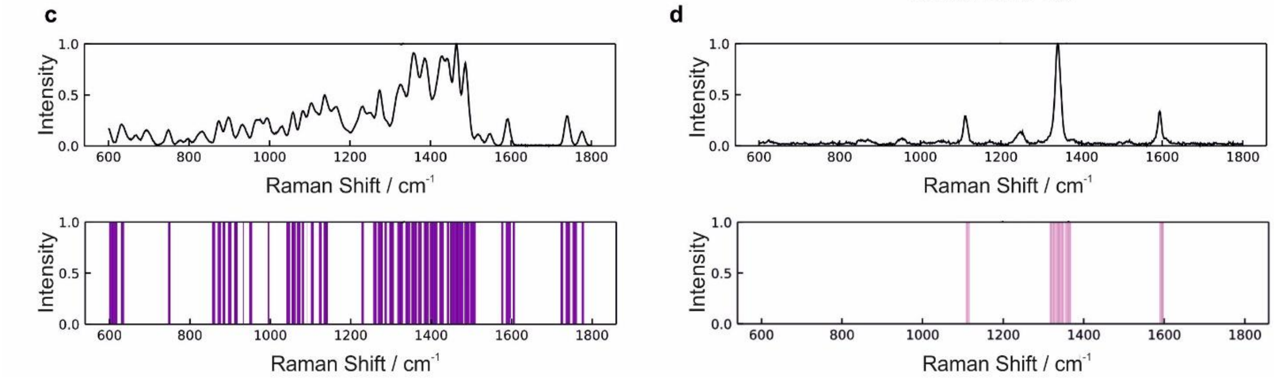

Figure S5. (a). Spiked plasma with TBI-indicative biomarkers where 10μL of analyte spiked the test sample matrix (50μL) and the response recovered in the assay by comparison to an identical spike in the standard diluent. 98.7±1.7% recovery within the spiked samples was observed. Standard SERS spectra of the pure **(b)** Fuc6GlcNAc, **(c, top)** Manα3 and **(d, top)** P-nitrophenyl galacto-N-bioside with the corresponding **(c-d, bottom)** specific fingerprints barcoded *via* the representative peaks of the highest intensity and spectral differences and used to identify the target analytes. Spike-and-recovery was used for validating and

assessing the assay and to establish whether there is a difference between the diluent used to prepare the standard curve and the biological sample matrix.

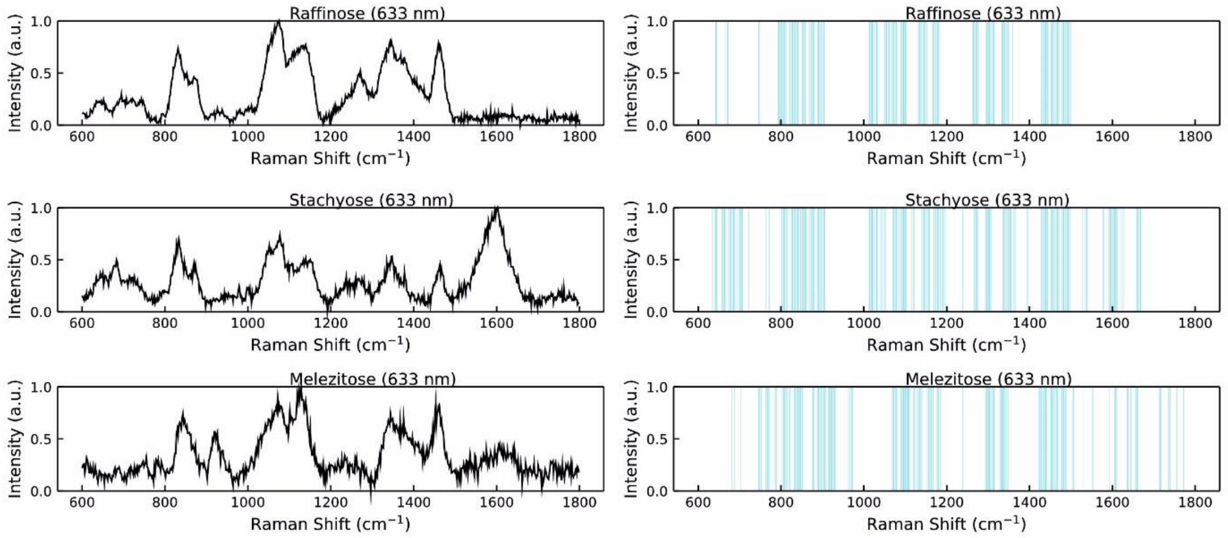

Figure S6. Representative average spectra of the pure raffinose, stachyose and melezitose **(left)** generated the specific fingerprints were barcoded *via* the representative SERS peaks of the highest intensity and spectral differences, subsequently used to identify the target analytes within the biomatrices.

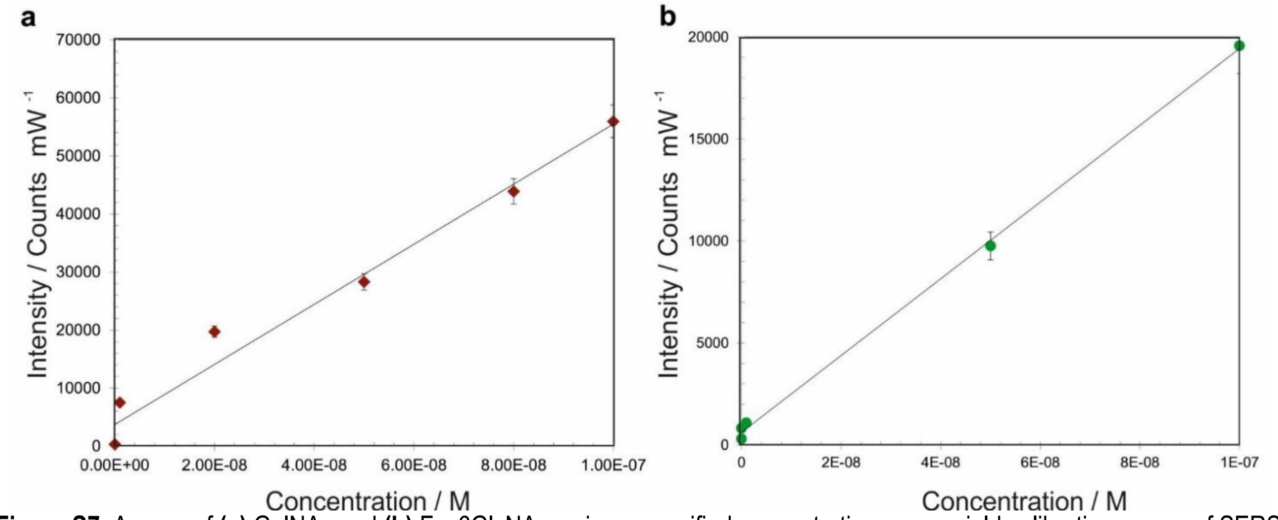

Figure S7. Assays of **(a)** GalNAc and **(b)** Fuc6GlcNAc, using a specified concentration range, yield calibration curves of SERS spectra acquired with at an excitation laser of 633nm. A good linear correlation was observed between glycan concentrations and SERS intensity with R^2=0.909 and 0.891 for GalNAc and Fuc6GlcNAc, respectively. Each point represents the mean ± s.d.e.v. of three measurements at each concentration used to calculate the LoD and LoQ.

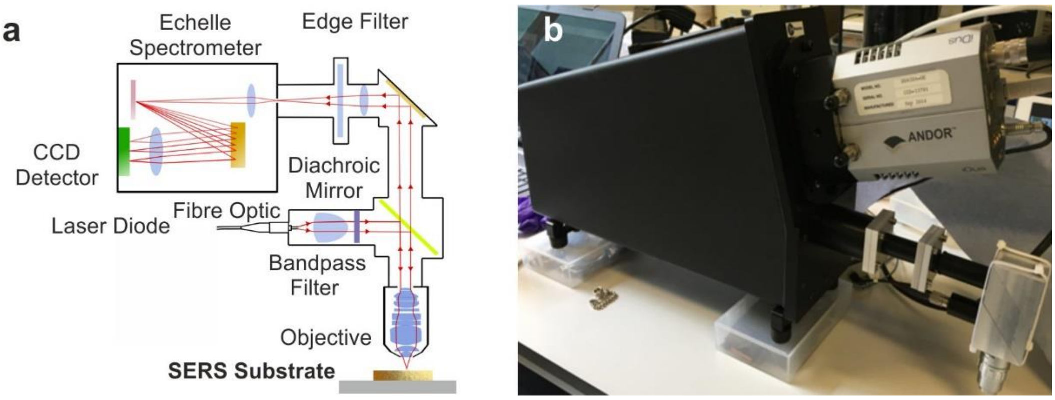

Figure S8. The home-built miniaturised spectrometer **(a)** uses a diode laser, fed down a fibre optic and passed through a collimating lens and subsequently, the beam is reflected from a dichroic mirror down through a 20x microscope objective onto the SERS substrate. The scattered beam is reflected through the microscope objective lens and passes through the dichroic mirror and onto a gold coated mirror that reflects it through a lens and focuses the beam onto the slit of the spectrometer. In

between the spectrometer slit and the focusing lens an edge filter is located to block the Rayleigh scattered light whilst allowing the longer wavelength light through. The different wavelengths of light are spread apart within the echelle spectrometer **(b)** and collected on the cooled CCD detector with the information being sent to a PC.

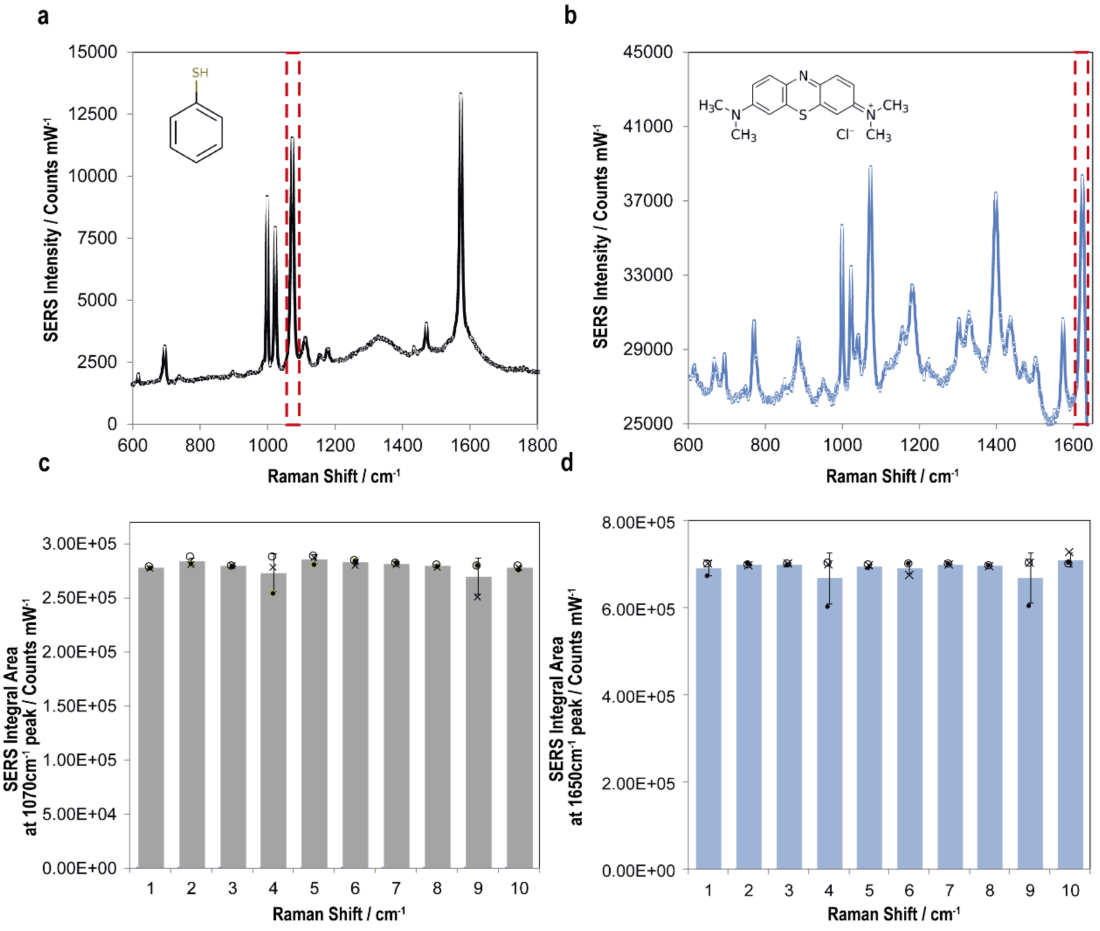

Figure S9. Reproducibility of the SERS Signals on the Electrohydrodynamically Generated Arrays. Representative SERS spectra of **(a)** benzenethiol (BT) and **(b)** methylene blue (MB) on uniform substrates across ten random locations on each substrate, for the highlighted peaks, at 10 s integration time, demonstrating reliable signal and substrate reproducibility. Repeatable SERS response was obtained from the EHD surfaces with relative standard deviation values of less than 8.5% and ~4.3% in the framework of one sample (error bars) and between the different samples (height of the bars), accordingly, for the two aforementioned Raman peaks (1070 cm^{-1} for BT and 1650 cm^{-1} for MB, respectively), comparable to the state-of-the-art SERS substrates. [1-2] **(c)** and **(d)** Intensities of the peaks at 1070 and 1650 cm^{-1} in the SERS spectra of BT and MB from 10 randomly selected positions on the gold-coated electrohydrodynamically fabricated pillars.

[1]. Liu X. *et* al. *Sci. Rep*. **2014**, 4, 5835
[2]. Huang J. *et* al. *Nano Lett.* **2013**, 13, 5039.
